# Exosomes derived from human umbilical cord MSCs rejuvenate aged MSCs and enhance their functions for myocardial repair

**DOI:** 10.1186/s13287-020-01782-9

**Published:** 2020-07-08

**Authors:** Ning Zhang, Jinyun Zhu, Qunchao Ma, Yun Zhao, Yingchao Wang, Xinyang Hu, Jinghai Chen, Wei Zhu, Zhongchao Han, Hong Yu

**Affiliations:** 1 Department of Cardiology, Second Affiliated Hospital, College of Medicine, Zhejiang University, 88 Jiefang Rd, Hangzhou, 310009 Zhejiang Province People’s Republic of China; 2 Cardiovascular Key Laboratory of Zhejiang Province, 88 Jiefang Rd, Hangzhou, 310009 Zhejiang Province People’s Republic of China; 3 Beijing Engineering Laboratory of Perinatal Stem Cells, Beijing Institute of Health and Stem Cells, Health & Biotech Co, Beijing, 100176 China

**Keywords:** Mesenchymal stem cells, Exosomes, Rejuvenation, MicroRNA, Myocardial infarction

## Abstract

**Background:**

Age and other cardiovascular risk factors have been reported to impair the activities of mesenchymal stem cells (MSCs), which will affect the efficacy of stem cell transplantation. The objective of the study is to investigate whether exosomes derived from human umbilical cord MSCs (UMSCs) could enhance the activities of bone marrow MSCs from old person (OMSCs), and improve their capacity for cardiac repair.

**Methods:**

Exosomes extracted from conditioned medium of UMSCs were used to treat OMSCs to generate OMSCs^Exo^. The key molecule in the exosomes that have potential to rejuvenate aged MSCs were screened, and the role of OMSC was tested in the mouse model of mycardial infarction (MI).

**Results:**

We found the activity of senescence-associated β-galactosidase and the expression of aging-related factors such as p53, p21, and p16 were significantly higher in OMSCs than those in UMSCs. After treatment with UMSC exosomes, these senescence phenotypes of OMSCs were remarkably reduced. The proliferation, migration, differentiation, and anti-apoptotic and paracrine effect were increased in OMSCs^Exo^. In vivo study, mice with cardiac infarction had significantly better cardiac function, less fibrosis, and more angiogenesis after they were injected with OMSCs^Exo^ as compared with those with OMSC. There was more miR-136 expression in UMSCs and OMSCs^Exo^ than in OMSCs. Upregulation of miR-136 by transfection of miR-136 mimic into OMSCs significantly attenuated the apoptosis and senescence of OMSCs. Apoptotic peptidase activating factor (Apaf1) was found to be the downstream gene that is negatively regulated by miR-136 via directly targeting at its 3′UTR.

**Conclusion:**

Our data suggest that exosomes from young MSCs can improve activities of aged MSCs and enhance their function for myocardial repair by transferring exosomal miR-136 and downregulating Apaf1.

## Background

Stem cell-based transplantation is one of the most promising strategies of regenerative medicine for the treatment of different diseases, such as cardiovascular diseases [1, 2]. At present, mesenchymal stem cells (MSCs) are the most used cell type for treating various diseases. Given that allogenic MSCs have the risk of immunogenicity, especially for the elderly patient, patient-derived autologous MSCs may be the safer choice in terms of avoiding unwanted immune responses [3, 4]. Unfortunately, age and other cardiovascular risk factors have been reported to reduce the availability of stem cells and to impair their function and activity [5, 6], thus limiting their therapeutic usefulness in the aged patients who could most benefit. Therefore, strategies to ameliorate the biological phenotype and function of MSCs from old people (OMSCs) are urgently needed to achieve higher therapeutic efficacy when autologous MSCs are used.

The current paradigm is that MSCs accomplish many of these therapeutical functions via a paracrine mechanism [7]. Paracrine effects are typically mediated by bioactive components that are present in secreted membrane vesicles [8]. Cellular senescence not only impacts the cell function, but also influence their neighboring cells through secreting high levels of inflammatory cytokines, chemokines, growth factors, and metalloproteinases, which is so-called senescence-associated secretory phenotype (SASP) [9, 10]. In contrast, young cells have been shown to be able to rejuvenate aged cells through secreting “young signals” [11]. Recent study showed that extracellular vesicles (EVs) derived from either induced pluripotent stem cells (iPSCs) or MSCs can alleviate senescence of human cells in vitro [12]. It suggests that paracrine factors of MSCs may play an important role in the anti-aging process. Thus, the EVs secreted from young cells appear to be a novel anti-aging factor. As a major part of EVs, exosomes become a research focus. Exosomes are biological nanovesicles of 30–120 nm in diameter and thought to be a potent secretory component of MSCs containing a wide range of contents including nucleic acids, proteins, lipids, mRNAs, and miRNAs [13, 14]. The paracrine effects exerted by human umbilical cord MSCs (UMSCs) are more intense than those exerted by bone marrow stem cells (BMSCs) and adipose-derived MSCs [15, 16]. However, the rejuvenation effect of exosomes secreted from UMSCs remains unclear and the mechanism remains to be clarified.

A growing number of studies have already confirmed that exosomal miRNAs play important roles in mediating intracellular cell communication. MicroRNAs are a class of small (18 ~ 22 nucleotides) single-stranded noncoding RNAs that suppress the expression of protein-coding genes by directing translational repression, mRNA degradation, or both. miRNAs mediate their regulatory action through imperfect binding to the 3′-untranslated region (3′-UTR) of target mRNAs carrying the complementary site [17]. Clinically, profiles in exosomes have been linked to disease pathologies including the aging process [18].

Here, we hypothesize that exosomes derived from UMSCs (Exo^UMSCs^) can rejuvenate OMSCs via transferring specific miRNAs, resulting in higher activities of OMSCs for myocardial repair. In this study, we found that Exo^UMSCs^ can ameliorate senescent phenotype and improve functions of OMSCs by releasing exosomal miR-136 which down-regulates apoptotic peptidase activating factor (Apaf1) in OMSCs. Such rejuvenated OMSCs can have better therapeutic effect for cardiac repair.

Our study sheds new light on the cell-free modification of aged stem cells as a potential therapeutic tool for tissue regeneration.

## Methods

### Human samples of umbilical cord blood and bone marrow

The research proposal for use of human samples was approved by the Second Affiliated Hospital Research Ethics Committee of Zhejiang University and in accordance with the 1964 Helsinki declaration and its later amendments or comparable ethical standards. Before samples were collected, all participants received and signed the written informed consent. Bone marrows from proximal femur of male patients at the age of 65 to 85 years were used. The human umbilical cord blood of newborn babies (*n* = 9) and the circulating blood of adult males (*n* = 29, age at 40–89 years old) were collected at the Second Affiliated Hospital of Zhejiang University (Hangzhou, China).

### Isolation and identification of MSCs

Human umbilical cord MSCs (UMSCs) were provided by National Engineering Research Center of Cell Products, State Key Laboratory of Experimental Hematology (Tianjin, China). Bone marrow MSCs were isolated from the bone marrow. Bone marrows obtained from the human proximal femur were washed with phosphate buffered saline (PBS) and separated by the isolation regents (Wealthlin Science and Technologies, Toronto, Canada) according to the user’s guide. The collected cells were seeded into 75 cm^2^ culture flasks with low glucose Dulbecco’s modified Eagle’s medium (DMEM) containing 10% (v/v) fetal bovine serum (FBS; Life Technologies) and 100 U/ml penicillin/streptomycin (v/v) (SP) in a humidified atmosphere with 95% air/5% CO_2_ at 37 °C. After 48–72 h culture, non-adherent cells were removed by changing the medium. The medium was changed every 2–3 days. Cells were passed when cells reached ~ 80% confluence and MSCs at passage 2–6 were used for the study. MSCs were characterized for their surface markers by a FACS Canto II Flow Cytometer (BD Bioscience, San Jose, CA, USA). Briefly, 1 × 10^6^ cells were collected and suspended in 100 μl PBS with 1% FBS, and then incubated with fluorescence-labeled antibodies against the surface markers (mesenchymal surface markers: PE-CD29, PE-105, APC-CD166; endothelial cell surface marker: FITC-CD34; hematopoietic surface marker: FITC-CD45 and isotype-matched control) for 1 h (Fig. S1B). After washed with PBS twice, the cells were analyzed for the cell surface markers using FACS via software (BD Bioscience).

### Exosomes isolation and identification

Exosomes were isolated using differential centrifugation according to the described methods. UMSCs (1 × 10^6^ cells) were seeded on the 75 cm^2^ culture flasks with DMEM containing 10% (v/v) FBS and 100 U/ml SP in a humidified atmosphere at 37 °C. Once cells were passed at 80–90% confluence (~ 1 × 10^7^ cells), UMSCs were washed with PBS twice and cultured in the same volume of DMEM with exosome-removed serum for 48 h. The supernatant was harvested and centrifuged at 300×*g* for 10 min and 10,000×*g* for 30 min to discard the dead cells. The supernatant was concentrated in a Concentrator with 100KD MW cutoff under centrifugation at 2500×*g* for 10 min for several times. The concentrated supernatant was then centrifuged at 100,000×*g* for 70 min to get exosome pellet which was resuspended in PBS and centrifuged again under 100,000×*g*. Lastly, exosomes in the pellet were resuspended in 100 μl PBS and stored at − 80 °C. A total 20 T75 flasks of UMSCs (~ 20 × 10^7^ cells) were used to achieve the final ~ 60 μg exosomes that were resuspended in 100 μl PBS. The morphology of exosomes was visualized by transmission electron microscope (TEM). The size distribution of exosomes was measured by dynamic light scattering (DLS) with a Nanosizer™ instrument (Malvern, UK).

### Animal experimental

Myocardial infarction (MI) model was established on 6–8-week male C57BL/6 mice. Thoracotomy was performed at the fourth intercostal space to expose the heart and left anterior descending coronary artery (LAD). The peri-infarct myocardial region was injected at five different points with a total 30 ul PBS with or without 1 × 10^6^ MSCs using a 30-gage needle. In the sham-operated mice, LAD was not ligated. The mice were randomly separated into 5 groups: sham, DMEM, OMSCs, OMSCs^Exo^, and UMSCs.

### miRNA mimic transfection

Cells (1 × 10^5^) were seeded in 12-well plates the day before transfection. The cells were transfected with 50 nM of miR-136 mimic and its non-specific miRNA negative control (Ambion, Austin, TX, USA), using Lipofectamine 3000 as the manufacture’s instruction (life science). MiR-136 expression in the transfected cells was determined by RT-qPCR at 48 h after transfection.

### Plasmid construction and luciferase activity assay

Luciferase activities were measured by a Dual-Glo Luciferase Reporter Assay Kit (Promega). To elucidate whether Apaf1 was a target gene of miR-136, TargetScan (http://targetscan.org) and miRTarBase (http://miRTarBase.mbc.nctu.edu.tw) were used to predict the target genes that may be regulated by miRNA molecules. Apaf1was identified as a potential target which can be regulated by miR-136. Bioinformatic analysis identified two putative binding sites of miR-136 at positions 138–145 and 1091–1098 of Apaf1 3′UTR. Wild-type (WT) and mutant binding regions of miR-136 in the 3′UTR of Apaf1 gene were cloned into pMIR-REPORT luciferase reporter plasmids (Invitrogen, USA). Four plasmids were generated: (1) WT, (2) mutation 1 at positions 138–145, (3) mutation 2 at positions 1091–1098, and (4) dual mutations at both sites of 3′UTR of Apaf1 gene. These plasmids were individually cotransfected with miR-136 mimic (100 nM; Sangon Biotech Co. Ltd., Shanghai, China) or negative control mimics into HEK293T cells (ATCC, Manassas, VA, USA). Renilla luciferase reporter plasmids were transfected as an internal positive control. After cultivation at 37 °C for 24 h, cells were assayed using the dual-luciferase assay system (Promega, Madison, USA) according to the manufacturer’s instructions. All assays were repeated at least three times.

### Statistical analysis

Data are presented as mean ± standard deviation (SD). All the data were analyzed with GraphPad Prism 6.02 software (San Diego, Calif). Continuous variables were compared by the Student *t* test. A comparison of more than two groups was performed by one-way ANOVA. A value of *P* < 0.05 was considered significant.

## Results

### MSCs from elderly are senescent and functional defective

**Fig. 1:**
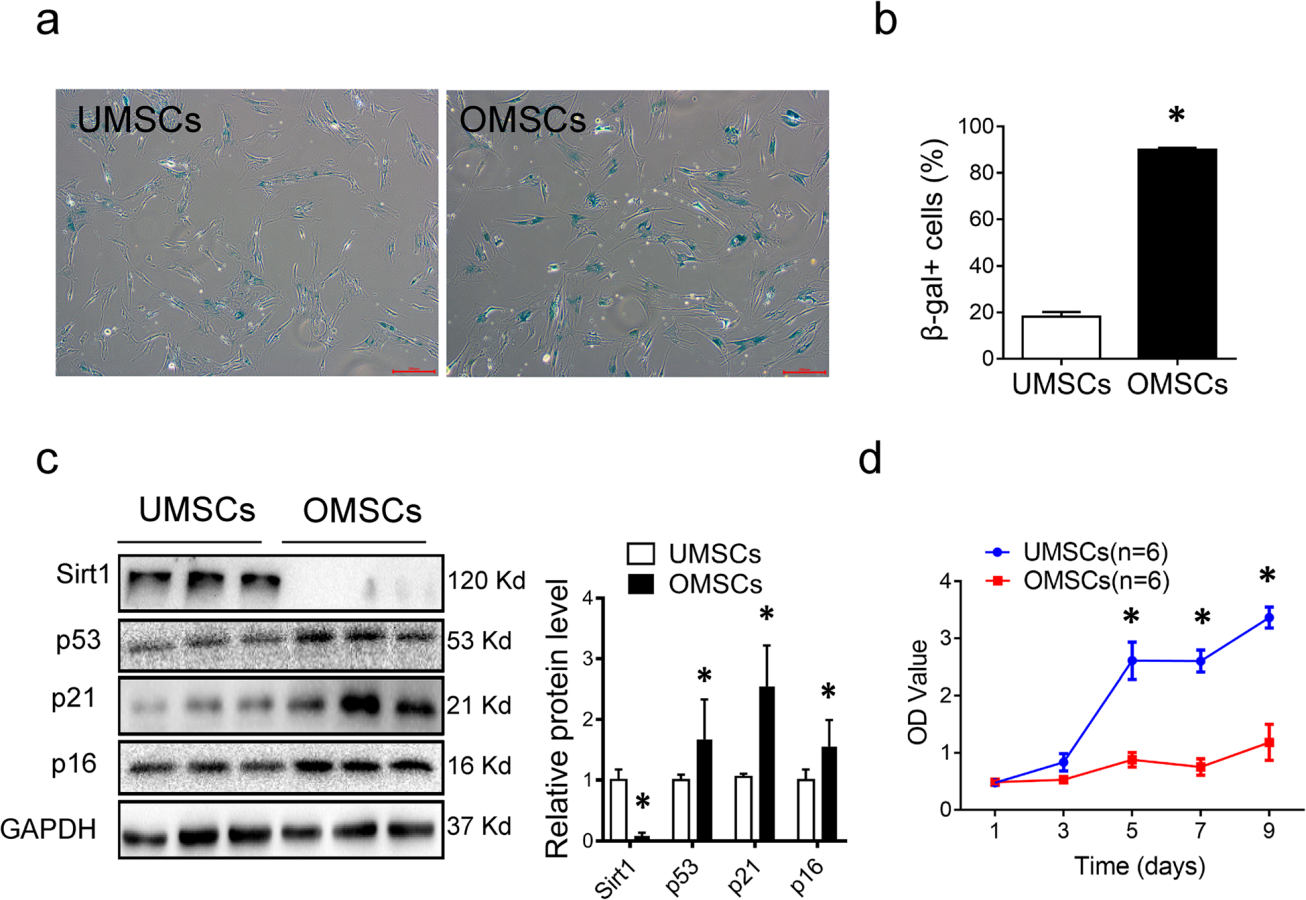
Characteristics of OMSCs and UMSCs. **a** OMSCs and UMSCs were stained for senescence associated β-galactosidase (β-gal) activity. Blue color indicates β-gal positive. **b** Quantification of β-gal-positive cells out of total cells in A. **c** Western blot analysis of aging-related protein markers (Sirt1, p53, p21, and p16). Quantification of specified proteins was shown in the bar graph. The values were normalized with GAPDH and then compared with that of UMSCs. **d** Time-course of cell growth determined by Cell Counting Kit-8 (CCK-8) assay was plotted for OMSCs and UMCSs. Each experiment was repeated three times. *P < 0.05 vs UMSCs

To understand the properties of MSCs from the elderly (OMSCs), we first compared senescent phenotypes of OMSCs (> 65 years old) with MSCs from the umbilical cord (UMSCs). As expected, more senescence-associated β-gal-positive cells were observed in OMSCs than in UMSCs (Fig. 1a, b). In line with these, the senescence-related markers p53, p21, and p16 were markedly upregulated and longevity marker Sirt1 was significantly decreased in OMSCs (Fig. 1c). Consistent with these results, the proliferation of OMSCs was significantly reduced as compared to that of UMSCs (Fig. 1d). Altogether, these data showed that OMSCs have significant senescent phenotypes, leading to a functional defection.

### Exosomes derived from UMSCs ameliorate senescent phenotype of OMSCs

**Fig. 2:**
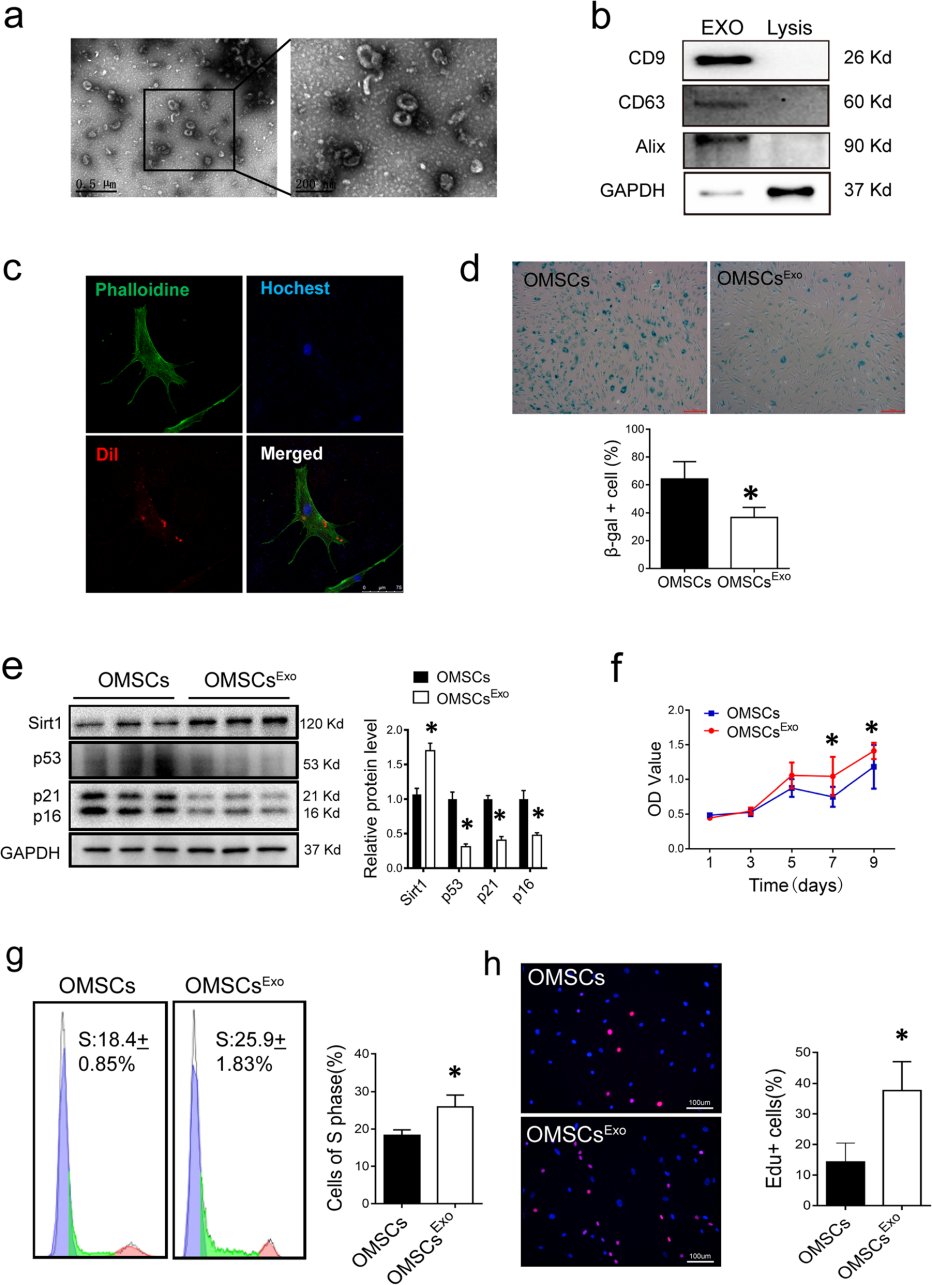
Exo^UMSCs^ mitigates the senescence phenotype of Old MSCs in vitro. **a** Representative images of exosomes derived from UMSCs (Exo^UMSCs^) under transmission electron microscope. Right panel is an amplified view of the selected area on the left. **b** Western blot analysis of exosomal specific markers (Alix, CD63, and CD9) for Exo^UMSCs^ (100 μg of exosomal protein was loaded), and cell lysis as negative control. **c** Representative fluorescence images of exosome internalization into OMSCs under confocal microscopy. Exo^UMSCs^ was marked with red florescence dye Dil and co-cultured with OMSCs. Red fluorescence represents exosomes uptaken by cells. **d** Representative images of β-gal staining for OMSCs with or without treatment with Exo^UMSCs^ and quantification of β-gal-positive cells out of total cells in A. **e** Western blot analysis age-related protein markers in OMSCs before and after treatment with Exo^UMSCs^. The proteins were quantified in the bar graph. **f** Time course of cell proliferation for OMSCs with or without treatment of Exo^UMSCs^ was analyzed by CCK-8 assay. **g** Flow cytometry analysis for cell cycle. Left side of peak in purple color represents cells in G1 phase, Middle part in green color represents cells in S phase, right peak in pink is the cells in G2 phase. **h** Edu staining images of OMSCs with or without treatment of Exo^UMSCs^. Pink represents Edu-positive cells. The nuclea of all cells were stained with Hochest showing blue. Scale bar, 100 μm. Edu^+^ cells out of total cells were quantified in bar graph. Each experiment was repeated three times. *P < 0.05 vs OMSCs

Exosomes from UMSCs (Exo^UMSCs^) were purified from the conditioned medium of UMSCs. The morphology of Exo^UMSCs^ was typical cup-shaped with double-layer membrane structure as visualized by transmission electron microscopy (TEM) (Fig. 2a). Western blot analysis confirmed that the isolated particles expressed exosome-specific markers: CD63, CD9, and Alixs (Fig. 2b). The particle size of Exo^UMSCs^ was around 60–150 nm as shown by dynamic light scattering (DLS) (Fig. S2). After OMSCs were incubated with Dil-labeled Exo^UMSCs^ for 48 h, fluorescence was detected inside the cells (Fig. 2c), indicating that Exo^UMSCs^ were efficiently internalized into the target cells. A number of β-gal-positive cells were significantly reduced in OMSCs after treated with Exo^UMSCs^ (Fig. 2d). Expression of aging-related factors p53, p21, and p16 was also markedly reduced, and the level of Sirt1 was increased in the Exo^UMSCs^-treated OMSCs (Fig. 2e). The growth rate of OMSCs was significantly increased (Fig. 2f), and more OMSCs entered into S phase of cell cycle after treatment with Exo^UMSCs^ (Fig. 2g). The numbers of EdU-positive OMSCs were significantly higher in the Exo^UMSCs^-treated OMSCs than the untreated cells (Fig. 2h). These results indicate that the exosomes secreted by UMSCs can rejuvenate the senescent MSCs.

### Exo^UMSCs^ renewed biological activities of OMSCs

**Fig. 3:**
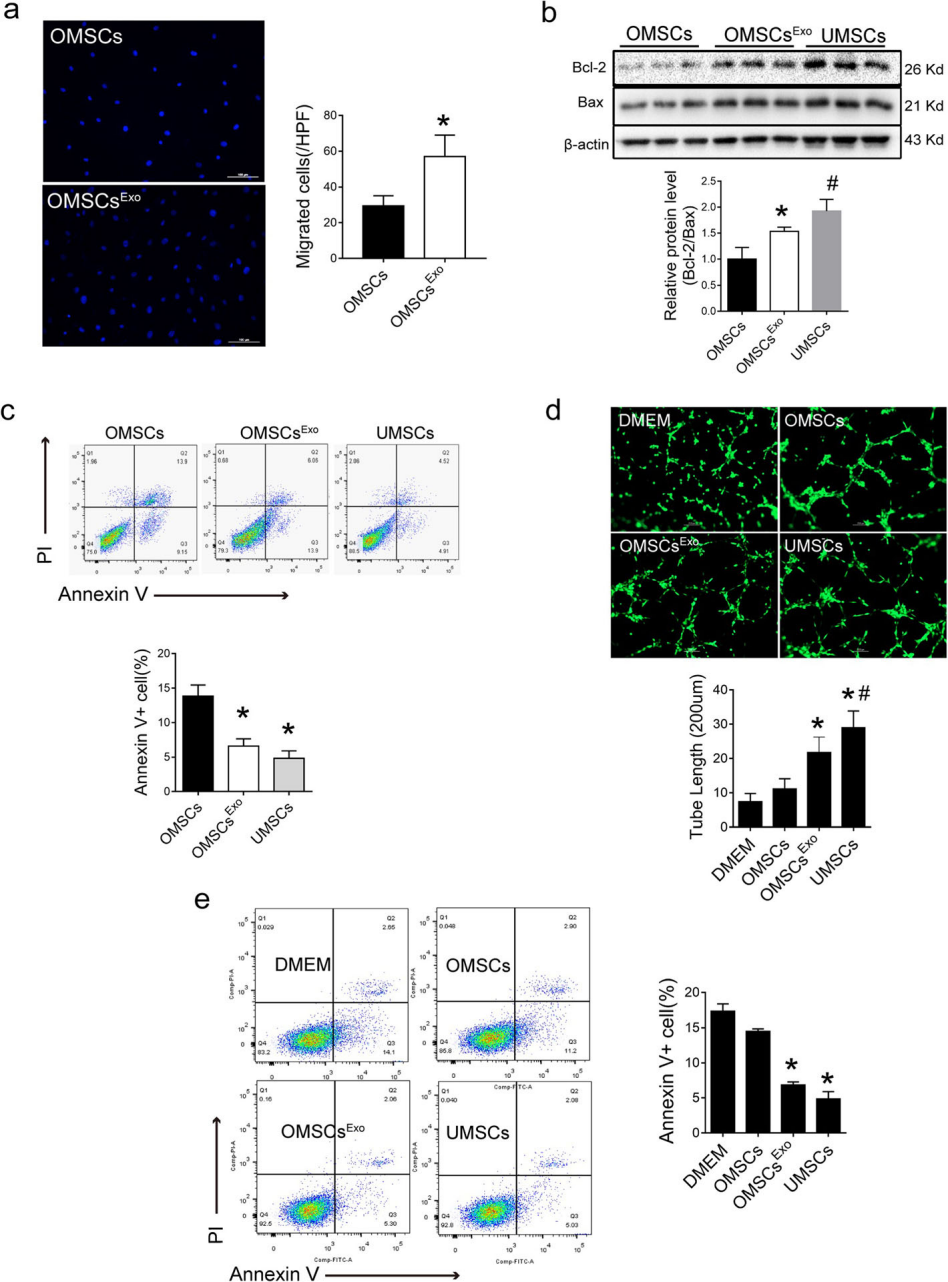
Exo^UMSCs^ promotes the biological function and paracrine effects of Old MSCs in vitro. **a** Mobility of OMSCs with or without treatment of Exo^UMSCs^ was analyzed by the Transwell assay. Migrated cells were visualized by stainings of Hochest for nuclear. Scale bar, 100 μm, and the quantification of the migrated cells in A. **b** Western blot analysis of Bcl2 and Bax for detection of cell apoptosis. β-actin served as loading control and the quantification of Bcl/Bax ratio in g. Each experiment was repeated three times. **c** Flow cytometry analysis of apoptosis and necrosis for specified cells after they were stained with Annexin V and Propidium Iodide (PI) and the quantification of cells in either early (Q3) or late (Q2) apoptosis. **d** Representative images of tube formation assay of HUVECs which were transduced with lentiviral vector carrying gene for GFP and cultured in DMEM alone or conditioned medium of specified cells (OMCs, OMSCs^Exo^, and UMSCs). Scale bar, 200 μm. **e** Quantification of tube length in A. **f** Flow cytometry analysis of apoptosis and necrosis in primary mouse cardiomyocytes after they were pretreated with DMEM or specified conditioned medium under hypoxia and serum deprivation condition for 24 h and then stained with Annexin V and PI. **g** Quantification of Annexin-V-positive cells (Q2 and Q3) in C. *P < 0.05 vs OMSCs; ^#^*P* < 0.05 vs OMSCs^Exo^

To examine if Exo^UMSCs^ could improve biological functions of OMSCs, OMSCs were pretreated with Exo^UMSCs^ for 48 h (OMSCs^Exo^). The migration capacity was remarkably increased in OMSCs^Exo^ measured by either Transwell assay (Fig. 3a) or scratch wound assay (Fig. S3a-d). The lower levels of Bcl-2/bax confirmed the decreased apoptosis of Exo^UMSCs^-treated OMSCs (Fig. 3b). Furthermore, a reduced number of apoptotic cells were observed in the Exo^UMSCs^-treated OMSCs (Fig. 3c) as compared with the untreated cells, and the rate of apoptotic cells was almost equivalent to that of UMSCs under the stressed condition. The differentiation potential of OMSCs into osteocytes, chondrocytes, and adipocytes was also increased after treated with Exo^UMSCs^ (Fig. S4).

Since the therapeutic effects of MSCs are primarily contributed by their paracrine functions, the effect of Exo^UMSCs^ on MSC paracrine functions was also studied. The conditioned media were collected from OMSCs, OMSCs^Exo^, and UMSCs to treat human umbilical vein endothelial cells (HUVECs) and primary mouse cardiomyocytes for assessing the pro-angiogenesis and anti-apoptosis effect of MSCs. Significantly increased tube formation was observed in HUVECs treated with the medium of OMSCs^Exo^ as compared with those treated with DMEM or medium of OMSCs (Fig. 3d). Similarly, the apoptosis rate of cardiomyocytes (CMs) under hypoxia and serum deprivation condition was attenuated after CMs were treated with the medium of OMSCs^Exo^ as compared with DMEM or OMSC groups, and reached a similar level as that of treated with the medium of UMSCs (Fig. 3e). These results suggested that exosomes secreted by UMSCs can ameliorate the biological function of OMSCs and enhance their paracrine effects.

### OMSCs pretreated with Exo^UMSCs^ have better cardioprotective effect in vivo

To investigate the effect of Exo^UMSCs^ on therapeutic functions of OMSCs for cardiac regeneration after myocardium infarction (MI), OMSCs or OMSCs^Exo^ treated with or without Exo^UMSCs^ were injected into myocardium immediately after MI. We measured cell survival after MSC transplantation in the heart tissue from different groups at day 7 after myocardial infarction, and found that the survival of UMSC and OMSC^Exo^ were significantly higher than those of the OMSC and DMEM groups(*P* < 0.05). There was an increased trend in survival rates of UMSC compared with OMSC^Exo^ (Fig. S5). We measured and analyzed the immune cells including CD3^+^ T cells, CD19^+^ B cells, Ly6G^+^ neutrophils, F4/80^+^ macrophages, and inflammatory factors such as IL-1b, Il-6, IL-12, TNFa, and MCP-1 in the heart tissue from different groups at day 7 after myocardial infarction. No differences in the amount of immune cells and inflammatory factors are detected between OMSC and UMSC groups (Fig. S6).

**Fig. 4:**
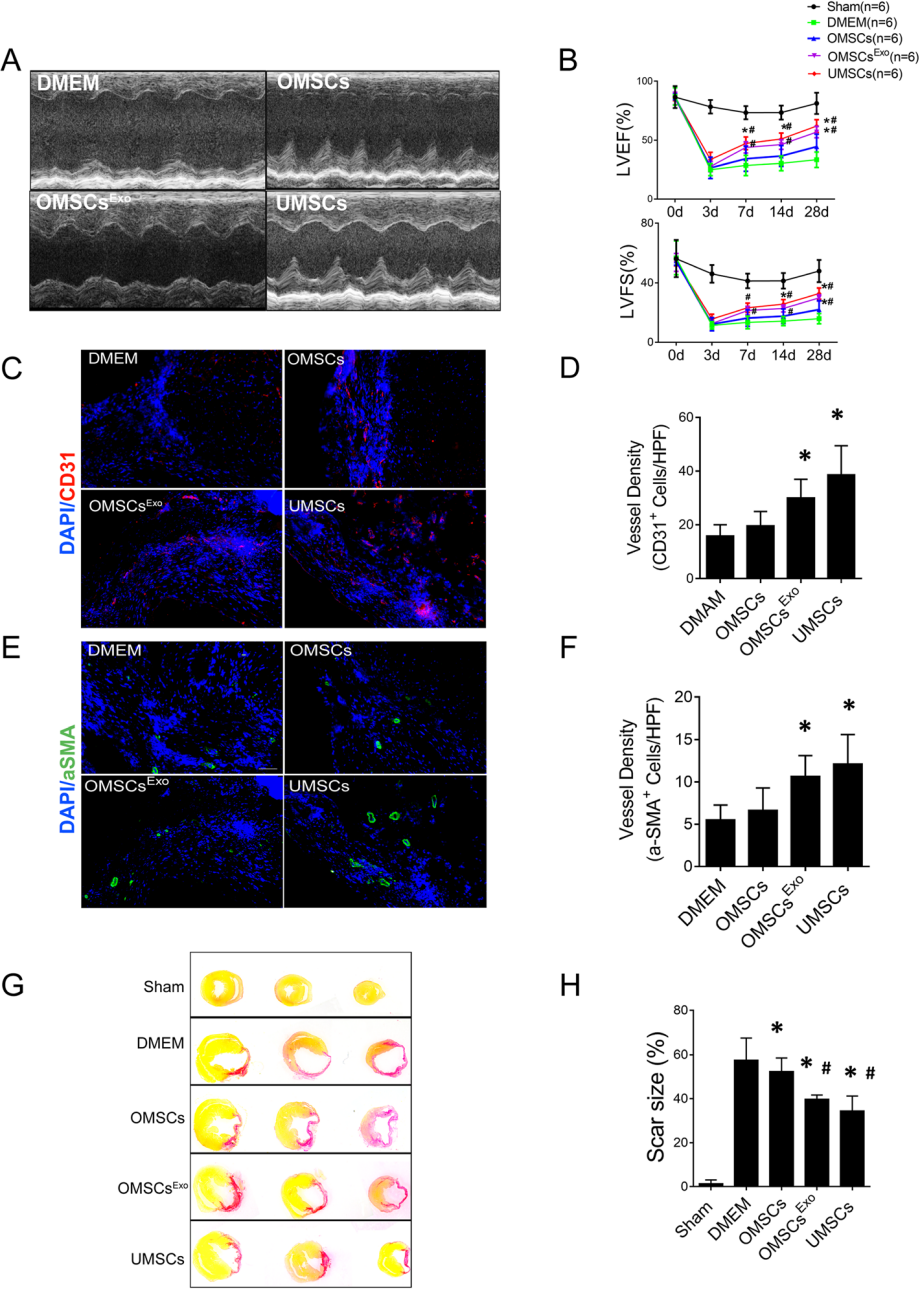
Effects of MSC transplantation on cardiac function after MI. **a** Representative echocardiographic images of specified mice at day 28 post MI. **b** Cardiac functions assessed by EF and FS (%) were obtained from echocardiographic images. **c** Representative images of immunofluorescence staining for ECs using Ab against CD31 (red) to illustrate angiogenesis in the border zone of ischemic hearts 28d post MI. **d** CD31-positive cells were quantified per high power field (HPF) to calculate the vessel density. **e** Representative images of immunofluorescence staining for vessel using antibody against α-SMA (green) to illustrate angiogenesis in the border zone of ischemic hearts 28 days post MI. **f** α-SMA-positive cells were quantified per HPF to calculate the mature vessel density. **g** Sections of hearts 28 days post MI were stained with Sirus red to illustrate the fibrosis (red) at infarct tissues. **h** The scar size was quantified and expressed as the ratio of the length of collagen deposited area (red) over perimeter of left ventricle. *n* = 6 hearts/group. Each experiment was repeated at least three times. *P < 0.05 vs DMEM; ^#^*P <* 0.05 vs OMSCs

Significant improvement in cardiac functions, both ejection fraction (EF%) and fractional shortening (FS%), was observed in the OMSCs^Exo^ group as compared with the OMSC group (EF 56 ± 5% vs 45 ± 12%, *p* < 0.05; FS 29 ± 3% vs 22 ± 7%, *p* < 0.05) (Fig. 4a), while OMSC treatment also resulted in certain improvement in cardiac function as compared with the control mice treated with DMEM (EF 45 ± 12% vs 34 ± 7%, *p* < 0.05; FS 22 ± 7% vs 16 ± 4%, *p* < 0.05). The cardioprotective effect of OMSCs^Exo^ reached close to that of UMSCs (Fig. 4b). The density of CD31^+^ capillary and vwF^+^ cells were significantly increased in mice injected with OMSCs^Exo^ (Fig. 4c, d and Fig. S7) as compared with those injected with OMSCs or DMEM 28 days after infarction. Similar trends were observed in arteriole density as measured by α-SMA staining (Fig. 4e, f). The scar size at 28 days after MI was significantly smaller in the OMSCs^Exo^ group than that of the OMSC and DMEM groups (Fig. 4g, h). These results indicate an improvement in therapeutic functions of OMSCs after treated with Exo^UMSCs^.

### miRNA-136 was a key effecter of Exo^UMSCs^ to rejuvenate OMSCs

**Fig. 5:**
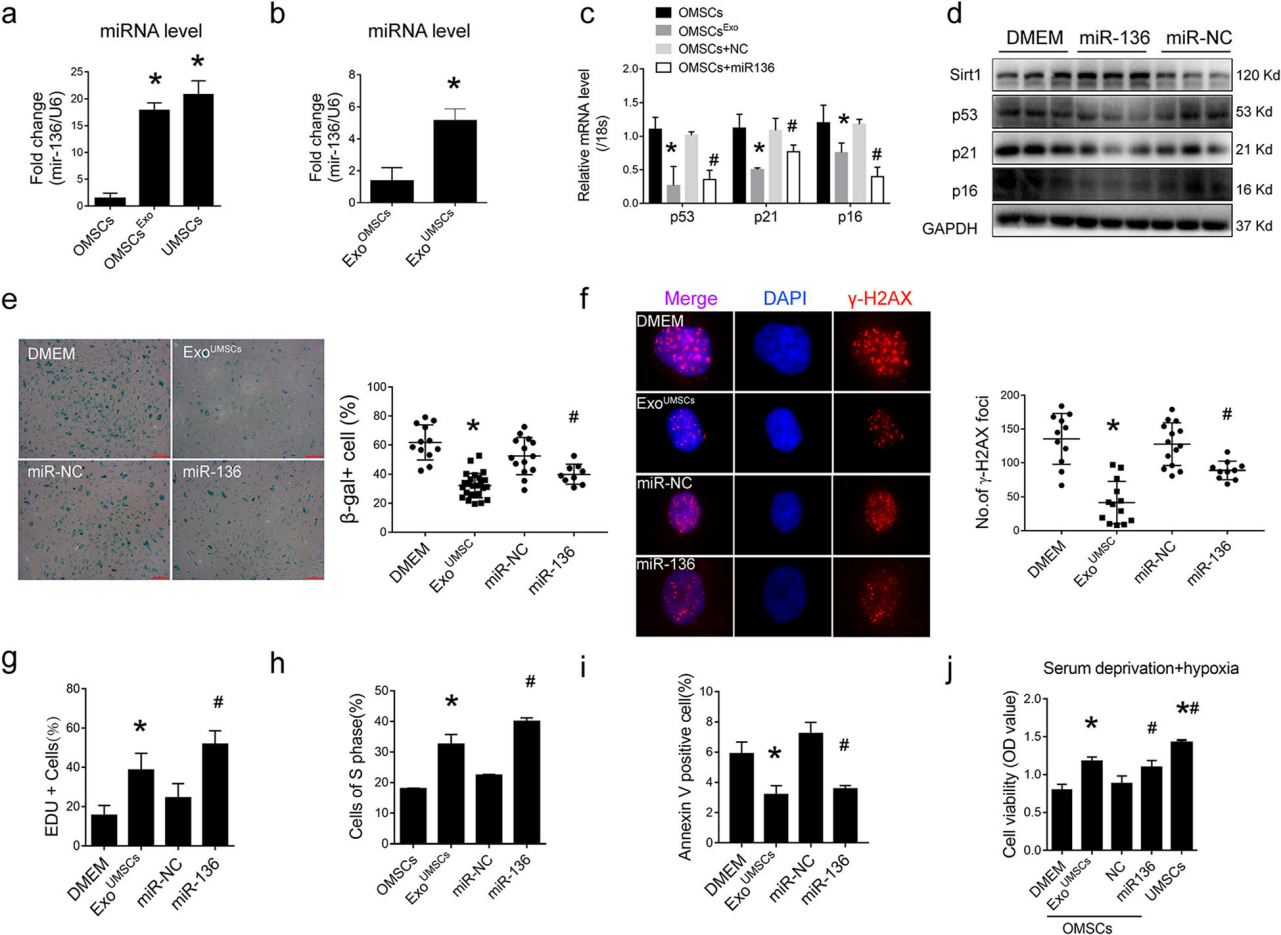
Effects of miR-136 on OMSCs. **a** Real-time RT-PCR analysis of miR-136 expression in UMSCs and OMSCs with or without treatment with Exo^UMSCs^. **b** Real-time PT-PCR analysis of miR-136 levels in exosomes derived from OMSCs and UMSCs. The expression level of miR-136 was normalized to U6. **c** Real-time RT-PCR analysis of p53, p21, and p16 expressions in OMSCs which were transfected with miR-136 mimic or scrambled miRNA (miR-NC), or treated with Exo^UMSCs^ for 48 h. **d** Western blot analysis of senescence-related proteins p53, p21, and p16 in OMSCs transfected with miR-136 mimic or miR-NC. GAPDH was used as a loading control. **e** Representative images of β-gal staining in OMSCs transfected with miR-136 mimic or miR-NC. β-gal-positive cells out of total cells were quantified in the bar graph. **f** Representative images of immunofluorescent-stained OMSCs which were transfected with miR-136 mimic or miR-NC, or treated with Exo^UMSCs^ for 48 h, and then stained with Ab against γ-H2AX (red) and DAPI (blue). Scale bar, 100 μm. Quantification of γ-H2AX foci in the specified cells. **g** Quantification of Edu-positive cells in OMSCs which were treated with Exo^UMSCs^, or transfected with miR-136 mimic or miR-NC. **h** Quantification of OMSCs at S phase of cell cycle as a percentage of total cells. OMSCs were cultured under the specified conditions and analyzed for their DNA contents using flow cytometry. **i** Quantification of apoptotic OMSCs which were treated with Exo^UMSCs^ or transfected with miR-136 mimic or miR-NC, and then cultured under hypoxia and serum deprivation conditions. Apoptosis of OMSCs was detected by Annexin V/PI staining. **j** Cell viability of OMSCs with indicated treatments were assessed by CCK-8 assay under serum deprivation under hypoxia condition. *P < 0.05 vs OMSCs/DMEM; **P < 0.05 vs OMSCs; ^#^*P <* 0.05 vs OMSCs + miR-NC/miR-136

To study the mechanism of Exo^UMSCs^-mediated rejuvenation of OMSCs, the profiles of age-related miRNAs were screened from database (Miranda and TargetScan) and literatures [19, 20] as most of the effects of exosome were carried out through exosomal miRNAs. Among these miRNAs, miR-136 was the most abundant in UMSCs, as compared with OMSCs, and was increased more than 20-fold in OMSCs after cultured with Exo^UMSCs^, whereas no significant difference was observed for other miRNAs such as miR-106a, miR-155, and miR-29C among these groups (Fig. 5a, Fig. S8). Moreover, the level of miRNA-136 in Exo^UMSCs^ was also higher than that in Exo^OMSCs^ (Fig. 5b). Next, we examined the role of miR-136 in regulating OMSC senescence and biological function. The mRNA and protein levels of senescent-associated markers p53, p21 and p16 (Fig. 5c, d), as well as the β-gal^+^ cells, were markedly reduced in OMSCs after transfected with miR-136 mimics (Fig. 5e). As a hallmark of cellular senescence and DNA damage, γH2AX-positive foci were enriched in OMSCs, as compared with UMSCs, and were remarkably decreased in miR-136 transfected OMSCs (Fig. 5f).

Furthermore, using an EdU incorporation assay to measure DNA synthesis, we found that the proliferation rate of OMSCs was also significantly increased after OMSCs were transfected with miR-136 mimic (Fig. 5g and Fig. S9a). More cells at S phase of cell cycle (Fig. 5h and Fig. S9b) and less apoptotic cells (Fig. 5i and Fig. S10a-c) were observed in the miR-136 overexpressed OMSCs in comparison with the original OMSCs. Exo^UMSCs^ and miR-136 mimics also significantly increased the cell survival rate (Fig. 5j) under hypoxia and serum deprivation condition (Fig. S11) mimicking the microenvironment of MI in vivo, and resulted in a similar viability as that of UMSCs.

### *Apaf1* is a functional direct target of miR-136 involving in OMSC survival

**Fig. 6:**
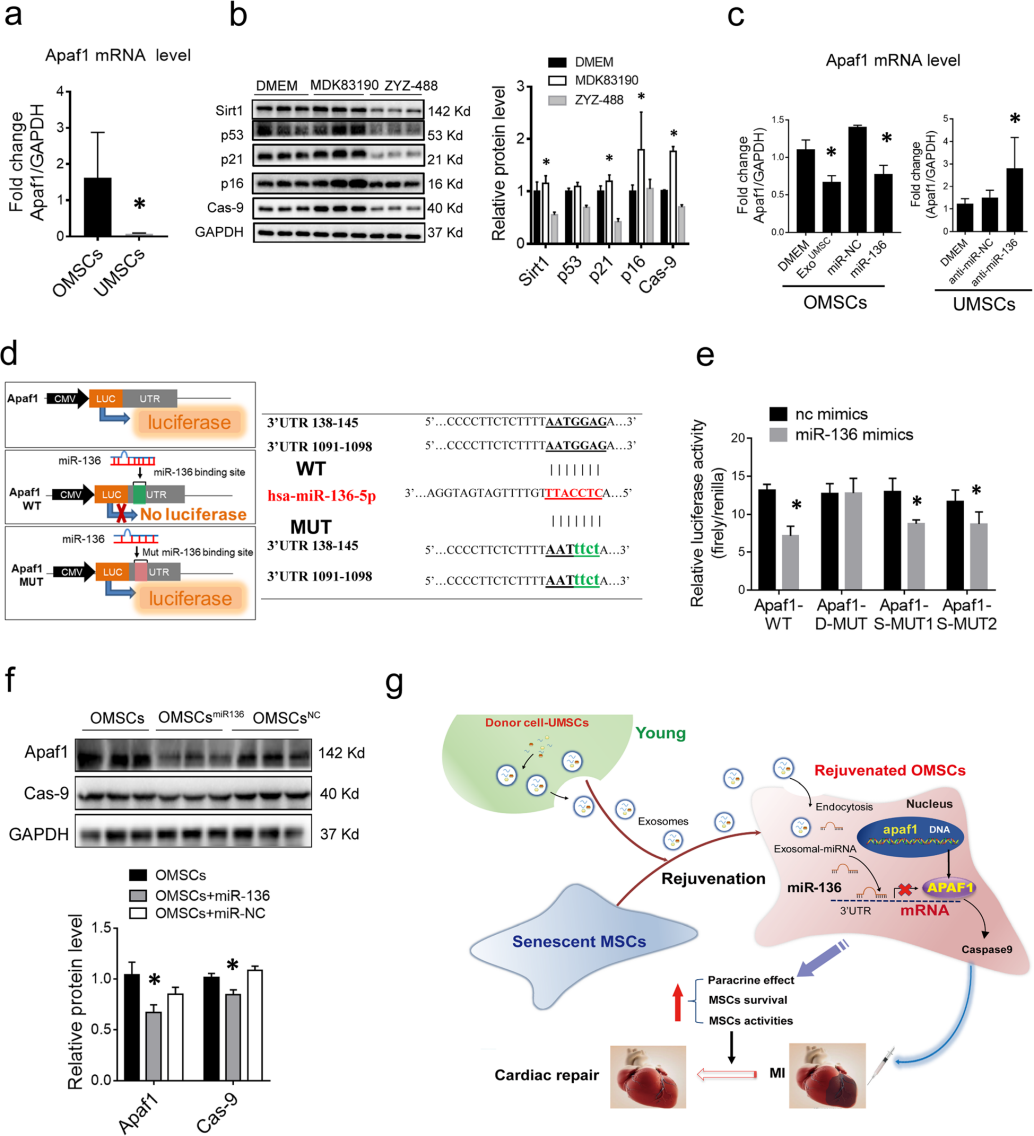
MiR-136 downregulates Apaf1 expression through directly binding with its noncoding region. **a** Real-time RT-PCR analysis of Apaf1 level in OMSCs and UMSCs, GAPDH used as control. **b**. Western blot analysis of senescence-related proteins p53, p21, and p16 in OMSCs treated with agonist (MDK83190) and inhibitor (ZYZ-488) of apaf1. GAPDH used as control. **c** Real-time RT-PCR analysis of Apaf1 level in OMSCs which were treated with Exo^UMSCs^ or transfected with miR-136 mimic or miR-NC for 48 h (left panel), or Apaf1 mRNA in UMSCs after the cells were transfected with miR-136 inhibitor (anti-miR-136) or anti-miR-NC for 48 h (right). **d** Apaf1 was predicted as the target mRNA of miR-136 by TargetScan (right). Schematic diagram showed the potential two binding sites for miR-136 on the 3′UTR (138–145) and (1091–1098) of Apaf1, and their designed mutants. Luciferase reporter vectors with different Apaf1 3′UTR (WT, double mutations at both sites (D-MUT), single mutation at either site (S-MUT1 and S-MUT2) were constructed (left). **e** 293 T cells were transfected with the specified luciferase reporter vector containing the WT, mutant plasmids S-MUT1, S-MUT2, and D-MUT, along with miR-136 mimic or miR-NC. After 24 h, cell lysates were prepared and subjected to determination of relative luciferase activity by normalization of firefly luciferase activity with *Renilla* luciferase activity. **f** Western blot analysis of Apaf1 and its downstream target caspase 9 protein in OMSCs which were transfected with miR-136 mimic or miR-NC. GAPDH used as control. **g** Schematic cartoon illustrating that exosomes derived from human umbilical cord MSCs rejuvenate aged MSCs and enhance their functions for myocardial repair. *P < 0.05 vs OMSCs/UMSCs+miR-NC; *P < 0.05 vs DMEM

To understand how miR-136 regulates functions of OMSCs, the downstream target of miR-136 in regulating cell senescence and survival was searched from literatures and confirmed via bioinformatic software (Miranda and miRtarbase). Apoptotic peptidase activating factor 1 (*Apaf1*) gene was found to be one of the predicted target genes for miR-136, and Apaf1 has been reported to play a crucial role in regulating cell survival and apoptosis [21]. The expression of *Apaf1* mRNA in OMSCs is higher than that in UMSCs (Fig. 6a). When OMSCs were treated with agonist (MDK83190) of Apaf1, the level of aging-related factors p53, p21, and p16 were upregulated and the level of Sirt1 was downregulated, whereas the inhibitor (ZYZ-488) of Apaf1 reversed this phenomenon (Fig. 6b). The data confirm that Apaf1 negatively affects cell aging.

We then examined the expression of *Apaf1* in OMSCs after treated with Exo^UMSCs^ or miR-136 mimic. Declined level of *Apaf1* mRNA was observed in Exo^UMSCs^-treated or miR-136-transfected OMSCs, as compared with untreated OMSCs or miR-NC transfected OMSCs, respectively (Fig. 6c). In agreement with this, *Apaf1* mRNA was increased in UMSCs after treated with miR-136 inhibitor. These results confirmed that the expression of *Apaf1* was negatively regulated by miR-136.

Two putative binding sites of miR-136 were identified at 3′UTR of *Apaf1* (positions 138–145 and 1091–1098) through bioinformatic analysis (Fig. 6d). To ascertain miR-136 directly binds to the 3′UTR of *Apaf1* and causes translational inhibition, such two predicted 3′UTRs of Apaf1 and their mutants were cloned into a luciferase reporter construct pmirGLO (Fig. 6d). The luciferase assay revealed that miR-136 mimic significantly reduced the activity of luciferase when the reporter gene was fused with the Apaf1 3′UTR, as compared with the miR-NC (Fig. 6e). The mutation of both putative miR-136 sites in the 3′UTR of *Apaf1* abrogated the effect of miR-136 on luciferase activity, while single mutation could not achieve such abrogation. Expressions of APAF1 protein and its downstream target caspase 9 were both suppressed in OMSCs after transfection with miR-136 mimic (Fig. 6f). These results indicated that Apaf1 is the downstream effecter of miR-136 through directly binding at the two sites of the 3′UTR region.

### More miR-136 in cord blood than in adult circulation

To investigate the expression of miR-136 during aging, blood from 29 healthy male adults (age > 40 years) and umbilical cord blood from 9 healthy maternity as the young group were collected, and the level of miR-136 in the plasma was measured by qRT-PCR. The expression level of miR-136 was significantly higher in cord blood than that in blood from adults (Fig. S12).

## Discussion

MSCs hold significant promise for regenerative medicine. Although transplantation of allogenic MSCs such as MSCs derived from perinatal tissues has been wildly accepted, infusion of allogenic MSCs from donors can often trigger immunoreaction, such as memory T cell response [22–24], whereas autologous MSCs can avoid such immune reaction since autologous MSCs have no or low immunogenicity [25]. However, aging and its associated diseases negatively impact MSC properties in terms of proliferation, paracrine function, and differentiation and impair their therapeutic effect when autologous MSCs are applied for old patients [26]. It has great clinical significance to restore the activity of aged MSCs for unrestricted stem cell transplantation. Physical [27], genetic, and biochemical [28] ways have been used to rejuvenate stem cells.

Here we found that exosomes derived from young MSCs (Exo^UMSCs^) ameliorate the senescence phenotype of OMSCs. The cell proliferation, mobility, and paracrine activity were significantly enhanced in OMSCs after Exo^UMSCs^ treatment. Such rejuvenated OMSCs^Exo^ had significantly increased activities in cardiac repair. Injection of OMSCs^Exo^ into infarct heart resulted in better cardiac function, more neovascularization, and less scar formation in comparison with that of OMSCs, which is consistent with the strengthened paracrine effects in vitro. More importantly, we for the first time identified the key factor achieving the rejuvenation: miR-136 and its target gene *Apaf1*. There are more miR-136 in both UMSCs and Exo^UMSCs^ than in OMSCs. After treatment with Exo^UMSCs^, the miR-136 level in OMSCs^Exo^ was significantly increased. Either Exo^UMSCs^ or miR-136 mimic can downregulate the expression of *Apaf1* in OMSCs, resulting in enhanced functions of OMSCs. These results suggest a novel approach to rejuvenate aged MSCs and renew their activities for tissue repair. The significance of this study is to expand the source of human MSCs and found a simple and safe alternative method to improve the therapeutic efficacy in cardiac disease. The novel method for rejuvenation of aged MSCs can also be expanded to broad clinical use for cell transplantation.

Exosomes are extracellular vesicles (EVs) with a size range of 30–120 nm and originate from the endosomal compartment where intraluminal vesicles within multivesicular bodies are released by exocytosis [29]. Exosomes can be secreted from almost all cell types and are found in most human biological fluids, including plasma, urine, synovial fluid, and bronchial lavage fluid [30]. Exosomes have emerged as novel and important players in intercellular communication, mainly through their ability to transfer biological content, consisting of proteins, mRNAs, miRNAs, and other non-coding RNAs, to recipient cell. Hence, exosomes have been identified as key mediators of paracrine effects, both in physiological and pathological states associated with aging. Recent researches have focused on exosomes as a powerful therapeutic tool in tissue repair and regeneration. Microvesicles from young MSCs were able to rejuvenate aged murine hematopoietic stem cells [31, 32]. Another research found that EVs secreted from human MSCs can reduce cellular ROS levels and alleviated aging phenotypes of senescent MSCs [33]. These studies have provoked great interest in examining the roles of EVs in cellular senescence and aging. However, these studies failed to illustrate the mechanism of such a rejuvenation effect of EVs.

In our study, we used human umbilical cord MSCs as a secretory source for the “young exosomes”. UMSCs have superiorities, including low immunogenicity, non-invasive procedure for harvest, and easy expansion in vitro, and have been used widely in tissue repair and regeneration. In comparison with MSCs derived from other human tissues such as the bone marrow and adipose, UMSCs were the best in terms of cell proliferation and paracrine effect and had the lowest expression of senescent markers: p53, p21, and p16. It has been reported that UMSC-derived exosomes activated several signaling pathways, which are conducive in cardioprotection [34]. Exo^UMSCs^ had also been used to rejuvenate the human skin [35].

Senescence has been initially characterized in vitro as a process limiting the proliferative capability of primary cells. The proliferation arrest, a main feature of cell senescence, is accompanied by changes in both morphology and function. Our study found that after Exo^UMSCs^ treatment, more cells entered into S phase and continued proliferation, while the aged MSCs were mostly in the stationary phase. Exo^UMSCs^ treatment reduced the senescence phenotypes and rejuvenated the activity of aged MSCs. In vivo, OMSCs^Exo^ were better than OMSCs in promoting neovascularization around the infarct zone, reducing cardiac fibrosis and preventing cardiomyocytes from apoptosis. The strengthened paracrine effect of OMSCs^Exo^ reached a similar level to that of UMSCs. Our work greatly expands the future application regimen of different human MSCs since rejuvenation by exosomes is a safer and more convenient way in comparison with other regents or genetic modification. However, it is still unknown if the functional improvement of aged stem cells is permanent by the exosomal treatment. Long-term effect of exosomes on MSCs is yet to be evaluated.

MicroRNAs (miRNAs) have been identified as the key post-transcriptional repressors of gene expression in various cells and participate in multiple biological processes, including aging and cell survival process [36]. Through direct exosomal transferring, miRNAs in the recipient cells were changed along with their functions [37]. However, early studies did not shed light on how EVs from human stem cells alleviate the aging phenotypes on senescent cells. Our current studies provided strong evidence for a new mechanism that UMSCs and their exosomes carry a high level of miR-136 which plays a major role for amelioration of aging phenotypes and viability when it was transferred into OMSCs.

MiR-136 was initially identified by cloning studies in mouse, and later verified in human embryonic stem cells [38]. Researches have shown that miR-136 acts as an anti-carcinoma miRNA in lung [39] and hepatocellular carcinoma [40], and involves the metastasis of colon cancer by suppressing epithelial-to-mesenchymal transition [41]. Here, we showed that both the cellular and exosomal level of miR-136 were higher in UMSCs than in OMSCs. After OMSCs were treated with Exo^UMSCs^ or transfected with miR-136 mimic, γH2AX foci, a marker of DNA damage and repair, was significantly reduced in OMSCs, and less cell apoptosis of OMSC^Exo^ was detected under stressed culture condition. We also unveiled the mechanism of miR-136 in the rejuvenation of OMSC^Exo^by identifying that miR-136 is a regulator of *Apaf1*. Luciferase assay and Western blot assay confirmed that miR-136 negatively regulates *Apaf1* expression via directly targeting its two sequences at 3′UTR.

The association between miR-136 and *Apaf1* has not been reported before. Especially, the role of miR-136/Apaf1 signaling in the regulation of senescence and activities of aged cells was unknown. Our results showed that UMSCs derived exosomes regulate aging-related phenotypes and functions in a miR-136/Apaf1-dependent way. Apaf-1 is a key molecule in the intrinsic pathway of apoptosis. It oligomerizes in response to cytochrome C release and forms a large complex known as apoptosome [42]. As the initiator caspase component of the apoptosome complex, procaspase-9 is recruited and activated by the apoptosome and leads to downstream caspase-3 processing [43]. It has been reported that Apaf-1 protein levels were controlled at post-transcriptional levels, which appears to be regulated at least to some extent by microRNAs, such as miR-23 and miR-27 in the central neural system [44]. In addition, co-transduction of Apaf-1 and caspase-9 triggers p53-dependent signaling pathways, and increased DNA damage contributing to cellular senescence was observed Casp 9- and Apaf1-deficient cells [45–47]. In our study, Apaf1 expression was inhibited by either Exo^UMSCs^ or miR-136 mimic. The level of caspase 9, a downstream target of Apaf1, was also declined. Besides its fundamental role in the apoptotic cell death pathways, Apaf-1 has a modulator effect on cell-cycle during DNA damage. Intriguingly, we found that there were more cells into S phase of cell-cycle after the cells were transfected with miR-136 mimics, which indicated that miR-136 and its downstream targets are involved in the cell proliferation and survival. In addition to apoptotic functions, non-apoptotic functions for Apaf-1 are emerging [48, 49]. Herein, mapping the interplay between these functions is essential for a complete understanding of the roles of Apaf-1 in health and aging diseases. Despite our novel finding of exosome-delivered miR-136 as a key element for rejuvenation, it remains unclear whether additional bioactive molecules (proteins, RNAs, lipids, etc.) are involved in the alleviation of senescence in OMCSs. It is undeniable that the effect of exosomes on aging cannot be achieved by a single miRNA.

In addition, we compared miR-136 level in the serum of the umbilical cord and adults, and found that miR-136 expression was higher in umbilical cord serum, which is consistent with the recent report about miRNA in circulating microvesicles as biomarkers for age-related cognitive decline [50]. Our results pave the way for the use of exosomal miR-136 as a biomarker for diagnosis.

## Conclusion

Our study reveals a new approach to reactive senescent cells through exosomes. We reported for the first time that exosomes secreted from young MSCs could effectively alleviate the senescent properties of aged MSCs and enhance their therapeutic effect. The key rejuvenation factor in Exo^UMSCs^ is miR-136 which directly binds to the 3′UTR of *Apaf1* mRNA and suppresses Apaf1 expression in the recipient cells. Rejuvenation of aged stem cells with an effective, convenient, and safe method could expand the availability of the donor for various applications (Fig. 6g). Our results point out miR136, as a key element released by young cells in the extracellular environment via exosomes, can improve surrounding cell viabilities, suggesting that we can reverse aging through miRNA gene editing. It is possible that epigenetic changes of aging hallmarks through exosomes or miRNAs could alter the physiological aging process and provide a key target for future rejuvenation.

## Supplementary information

**Additional file 1: Table S1.** Name and sequence of primers sets for real-time RT-PCR

**Additional file 2: Table S2.** Echocardiographic parameters of mice with indicated treatment (28d post- MI).

**Additional file 3: Table S3.** Construction of four luciferase reporter plasmids with different 3′UTR binding sites of Apaf1 gene.

**Additional file 4: Figure S1.** Characteristics and identification of OMSCs and UMSCs. Morphology of UMSCs and OMSCs were observed under microscope. MSCs were identified by flow cytometry with positive for cell surface markers CD29, CD44, CD90, and negative for CD34 (endothelial cell marker) and CD45 (hematopoietic marker). Differentiation of OMSCs into three lineages (osteogenesis, adipogenesis and chondrogenesis) was induced and visualized by alizarin red staining, oil red O staining, and toluidine blue staining, respectively.

**Additional file 5: Figure S2.** Characterization and Identification of exosomes derived from UMSCs. Representative images of Size distribution range (50-150 nm) of Exo^UMSCs^ was assessed by DLS analysis.

**Additional file 6: Figure S3.** Migration assay of MSCs. Mobility of OMSCs with or without treatment of Exo^UMSCs^ was analyzed by the transwell assay. Migrated cells were visualized by crystal violet staining. Scratch wound assay was conducted for assessing the migration potential of indicated cells.

**Additional file 7: Figure S4**. Differentiation potential of OMSCs after treatment with Exo^UMSCs^. Representative images of differentiation of OMSCs and OMSCs pretreated with Exo^UMSCs^ into osteocytes, adipocytes and chondrocytes, which was visualized by staining with alizarin red, oil red O, and toluidine blue, respectively.

**Additional file 8: Figure S5**. Cell survival after transplantation after MI. Flow cytometric analysis of Dil positive MSCs injected in the peri-infarct myocardial region after myocardial infarction in different groups.

**Additional file 9: Figure S6.** Immune cells and inflammatory factors expression after MSCs transplantation. Flow cytometric analysis of immune cells including CD3 + B cells, CD19 + T cells, Ly6G + neutrophils and F4/80 + macrophages in heart tissue at day 7 after myocardial infarction in different groups. RT-PCR analysis of inflammatory factors such as IL-1b, Il-6, IL-12, TNFa and MCP-1 in heart tissue at day 7 after myocardial infarction in different groups.

**Additional file 10: Figure S7.** Effects of MSCs Transplantation on angiogenesis after MI. Representative images of immunofluorescence staining for arterioles using shp against vwF (red) to illustrate matured vessel in the hearts. Scale bar,100 μm. vwF positive cells were quantified per HPF to calculate the matured vessel density in a bar graph.

**Additional file 11: Figure S8.** MiRNAs expression in MSCs. The expressions of miR-17, 19, 20a, 106a, 29, 136, and 155 in OMSCs, OMSCs treated with ExoUMSCs, and UMSCs were assessed by RT-qPCR. U6 was used as an internal reference gene.

**Additional file 12: Figure S9.** Detection of OMSCs proliferation and cell cycle after treatment with ExoUMSCs or miR-136. OMSCs which treated with Exo^UMSCs^, or transfected with miR-136 mimic or miR-NC were analyzed for cell cycle by EdU staining kit and flow cytometry analysis.

**Additional file 13: Figure S10.** Apoptosis of OMSCs after transfection of miR-136 or treatment with Exo^UMSCs^. apoptotic OMSCs were detected by Annexin V/PI staining and TUNEL staining which treated with Exo^UMSCs^ or transfected with miR-136 mimic or miR-NC, and then cultured under hypoxia and serum deprivation conditions.

**Additional file 14: Figure S11.** Quantification of viability of OMSCs. Cell viability of OMSCs with specified treatments was assessed by CCK-8 assay under serum deficiency.

**Additional file 15: Figure S12.** More miR-136 in cord blood than in adult circulation. Quantification of miR-136 level in serum from adults (*n* = 29) and cord blood of healthy maternity (*n* = 9) by real time PCR. U6 was used as an internal reference gene for miRNA.

## Data Availability

The datasets generated and/or analyzed during the current study are available in the Mendeley repository: Zhang, Ning; Zhao, Yun; Zhu, Jinyun; Ma, Qunchao; Wang, Yingchao; Hu, Xinyang; Zhu, Wei; Han, Zhongchao; Yu, Hong (2019), “figshare”, Mendeley Data, V4, http://dx.doi: 10.17632/fp4njbzdwt.4

## Abbreviations

BMSCs: Bone marrow stromal stem cells
FBS: Fetal bovine serum
UMSCs: Mesenchymal stem cells
OMSC: Mesenchymal stem cells from old person
Apaf1: Apoptotic peptidase activating factor
SASP: Senescence-associated secretary phenotype
EVs: Extracellular vesicles
TEM: Transmission electron microscope
DLS: Dynamic light scattering
MI: Myocardial infarction
LAD: Left anterior descending coronary artery
3′UTR: 3′-untranslated region
Exo^MSCs^: Exosomes from mesenchymal stem cells
HUVEC: Human umbilical vein endothelial cell
MSCs^Exo^: Mesenchymal stem cells were pretreated with exosomes
vWF: von Willebrand Factor
CM: Cardiomyocyte
EF: Ejection fraction
FS: Fractional shortening
DMEM: Dulbecco’s modified Eagle’s medium
