# Supplementary material for: Exosomes derived from human umbilical cord MSCs rejuvenate aged MSCs and enhance their functions for myocardial repair
Source: Stem Cell Res Ther. 2020 Jul 8;11:273. doi: 10.1186/s13287-020-01782-9 (PMC7346506; doi:10.1186/s13287-020-01782-9)
Supplement: Supplementary file 1 — Additional file 1: Table S1. Name and sequence of primers sets for real-time RT-PCR [file 13287_2020_1782_MOESM1_ESM.docx]

**Table S1. Name and sequence of primers sets for real-time RT-PCR**

| Gene name | Primer sequence |
| --- | --- |
| P53 | Forward：GGAAGTCCTTTGCCCTGAACT |
|  | Reverse： GTCTTCAGGTAGCTGGAGTGAG |
| P21 | Forward：TGTCCGTCAGAACCCATGC |
|  | Reverse： AAAGTCGAAGTTCCATCGCTC |
| P16 | Forward：ATGGAGCCTTCGGCTGACT |
|  | Reverse： GTAACTATTCGGTGCGTTGGG |
| Apaf1 | Forward：AAGGTGGAGTACCACAGAGG |
|  | Reverse：TCCATGTATGGTGACCCATCC |
| GAPDH | Forward：GGAGCGAGATCCCTCCAAAAT |
|  | Reverse： GGCTGTTGTCATACTTCTCATGG |
| Hsa-mir-136 | Forward: ACACTCCAGCTGGGACTC |
|  | Reverse：TGGTGTCGTGGAGTCG |
| Hsa-mir-155 | Forward: TGCCTCCAACTGACTCCTAC |
|  | Reverse: GCGAGCACAGAATAATACGTA |
| Hsa-mir-29c | Forward: TGCCAGGAGCTGGTGATTTCCT |
|  | Reverse: ACGGGCGTACAGAGGATCCCC |
| Hsa-mir-17 | Forward: ACACTCCAGCTGGGCAA-AGTGCT |
|  | Reverse: TGGTGTCGTGGAGTCG |
| Hsa-mir-19 | Forward: TGC GGTTCACAGTGGCTAAG |
|  | Reverse: CCAGTGCAGGGTCCGAGGT |
| Hsa-mir-20a | Forward: GCTAAAGTGCTTATAGTG |
|  | Reverse: GACTGTTCCTCTCTTCCTC |
| Hsa-mir-106a | Forward: GGAAAAGTGCTTACAGTGCAGGTAG |
|  | Reverse: GCACTGGATACGACAAAATATGGAAC |
| U6 | Forward：CTCGCTTCGGCAGCACA |
|  | Reverse：AACGCTTCACGAATTTGCGT |
