# Supplementary material for: Exosomes derived from human umbilical cord MSCs rejuvenate aged MSCs and enhance their functions for myocardial repair
Source: Stem Cell Res Ther. 2020 Jul 8;11:273. doi: 10.1186/s13287-020-01782-9 (PMC7346506; doi:10.1186/s13287-020-01782-9)
Supplement: Supplementary file 2 — Additional file 2: Table S2. Echocardiographic parameters of mice with indicated treatment (28d post- MI). [file 13287_2020_1782_MOESM2_ESM.docx]

**Table S2. Echocardiographic parameters of mice with indicated treatment (28d post-MI)**

| Groups | **Sham** | **DMEM** | **OMSCs** | **OMSCs^Exo^** | **UMSCs** |
| --- | --- | --- | --- | --- | --- |
| LVEF(%) | 81.33±9.01 | 34.12±7.17 | 44.93±11.77* | 55.98±4.68*# | 61.59±5.84*# |
| LVFS(%) | 49.97±11.43 | 16.25±3.76 | 22.23±6.70 | 28.70±2.86*# | 32.40±4.07*# |
| LVIDd(mm) | 2.90±0.23 | 4.53±0.67 | 4.00±0.64 | 3.74±0.36* | 3.49±0.35* |
| LVIDs(mm) | 1.46±0.38 | 3.73±0.83 | 3.14±0.76 | 2.67±0.33* | 2.36±0.30* |

LVEF, left ventricular ejection; LVFS, left ventricular fractional shortening; LVIDd: left ventricular internal diameters at end-diastole; LVIDs: left ventricular internal diameters at end-systole. **P<0.05* vs. DMEM; ^#^ *P<0.05* vs. OMSCs.
