# Supplementary material for: Exosomes derived from human umbilical cord MSCs rejuvenate aged MSCs and enhance their functions for myocardial repair
Source: Stem Cell Res Ther. 2020 Jul 8;11:273. doi: 10.1186/s13287-020-01782-9 (PMC7346506; doi:10.1186/s13287-020-01782-9)
Supplement: Supplementary file 3 — Additional file 3: Table S3. Construction of four luciferase reporter plasmids with different 3′UTR binding sites of Apaf1 gene. [file 13287_2020_1782_MOESM3_ESM.docx]

**Table S3. Construction of four luciferase reporter plasmids with different 3’-UTR binding sites of Apaf1 gene.**

| Plasmids | 3’UTR | Category | Sequences |
| --- | --- | --- | --- |
| WT | 138-145 | WT | 5’…CCCCTTCTCTTTT**AATGGAG**A…3’ |
|  | 1091-1098 | WT | 5’…CCCCTTCTCTTTT**AATGGAG**A…3’ |
|  |  |  | ▏▏▏▏▏▏▏ |
|  | hsa-miR-136-5p |  | 3’…AGGTAGTAGTTTTGT**TTACCTC**A…5’ |
| D-MUT | 138-145 | Mutant | 5’…CCCCTTCTCTTTT**AATttct**A…3’ |
|  | 1091-1098 | Mutant | 5’…CCCCTTCTCTTTT**AATttct**A…3’ |
|  |  |  | ▏▏▏▏▏▏▏ |
|  | hsa-miR-136-5p |  | 3’…AGGTAGTAGTTTTGT**TTACCTC**A…5’ |
| S-MUT1 | 138-145 | Mutant | 5’…CCCCTTCTCTTTT**AATttct**A…3’ |
|  | 1091-1098 | WT | 5’…CCCCTTCTCTTTT**AATGGAG**A…3’ |
|  |  |  | ▏▏▏▏▏▏▏ |
|  | hsa-miR-136-5p |  | 3’…AGGTAGTAGTTTTGT**TTACCTC**A…5’ |
| S-MUT2 | 138-145 | WT | 5’…CCCCTTCTCTTTT**AATGGAG**A…3’ |
|  | 1091-1098 | Mutant | 5’…CCCCTTCTCTTTT**AATttct**A…3’ |
|  |  |  | ▏▏▏▏▏▏▏ |
|  | hsa-miR-136-5p |  | 3’…AGGTAGTAGTTTTGT**TTACCTC**A…5’ |

WT: wild type 3’UTR; D-MUT: double mutations at both sites; S-MUT1 and S-MUT2: single mutation at 138-145 site and 1091-1098 site, respectively.
