# Supplementary figures and images for: Exosomes derived from human umbilical cord MSCs rejuvenate aged MSCs and enhance their functions for myocardial repair
Source: Stem Cell Res Ther. 2020 Jul 8;11:273. doi: 10.1186/s13287-020-01782-9 (PMC7346506; doi:10.1186/s13287-020-01782-9)

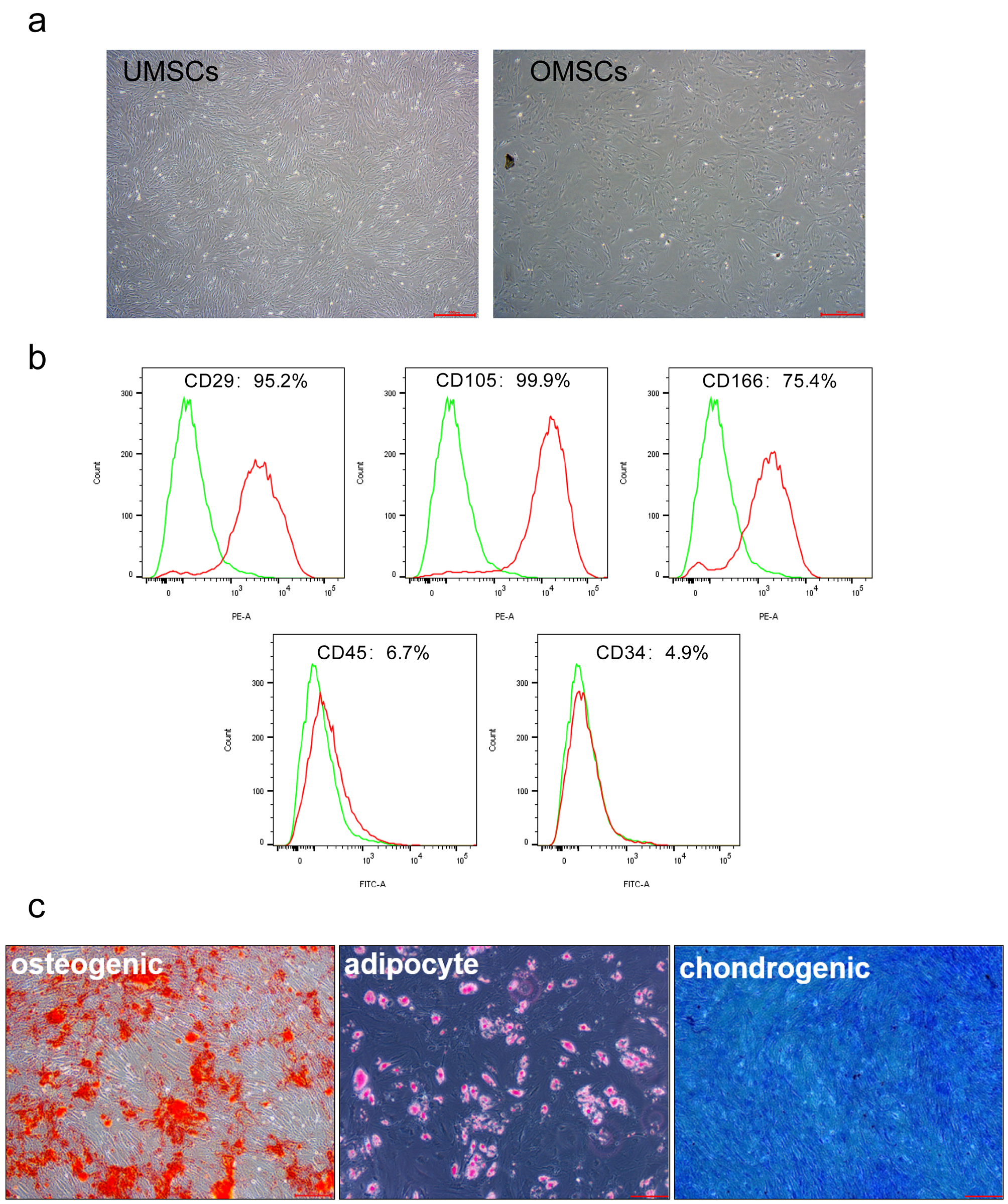

Supplement: Supplementary file 4 — Additional file 4: Figure S1. Characteristics and identification of OMSCs and UMSCs. Morphology of UMSCs and OMSCs were observed under microscope. MSCs were identified by flow cytometry with positive for cell surface markers CD29, CD44, CD90, and negative for CD34 (endothelial cell marker) and CD45 (hematopoietic marker). Differentiation of OMSCs into three lineages (osteogenesis, adipogenesis and chondrogenesis) was induced and visualized by alizarin red staining, oil red O staining, and toluidine blue staining, respectively. [file 13287_2020_1782_MOESM4_ESM.tif]

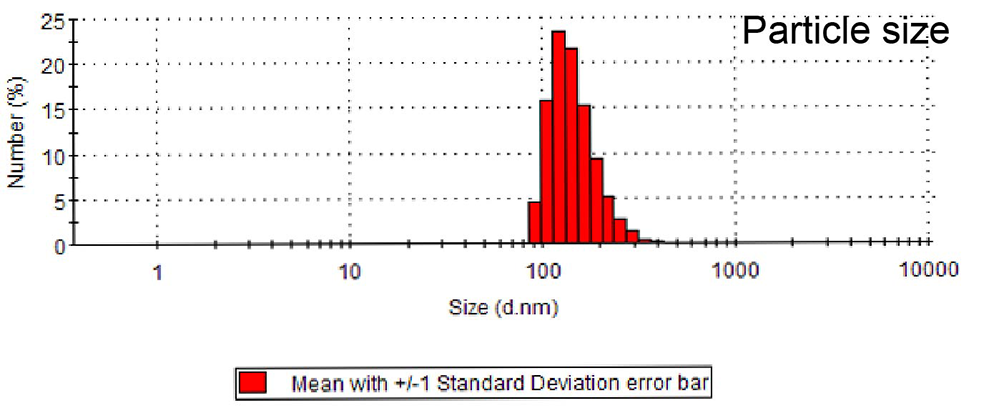

Supplement: Supplementary file 5 — Additional file 5: Figure S2. Characterization and Identification of exosomes derived from UMSCs. Representative images of Size distribution range (50-150 nm) of ExoUMSCs was assessed by DLS analysis. [file 13287_2020_1782_MOESM5_ESM.tif]

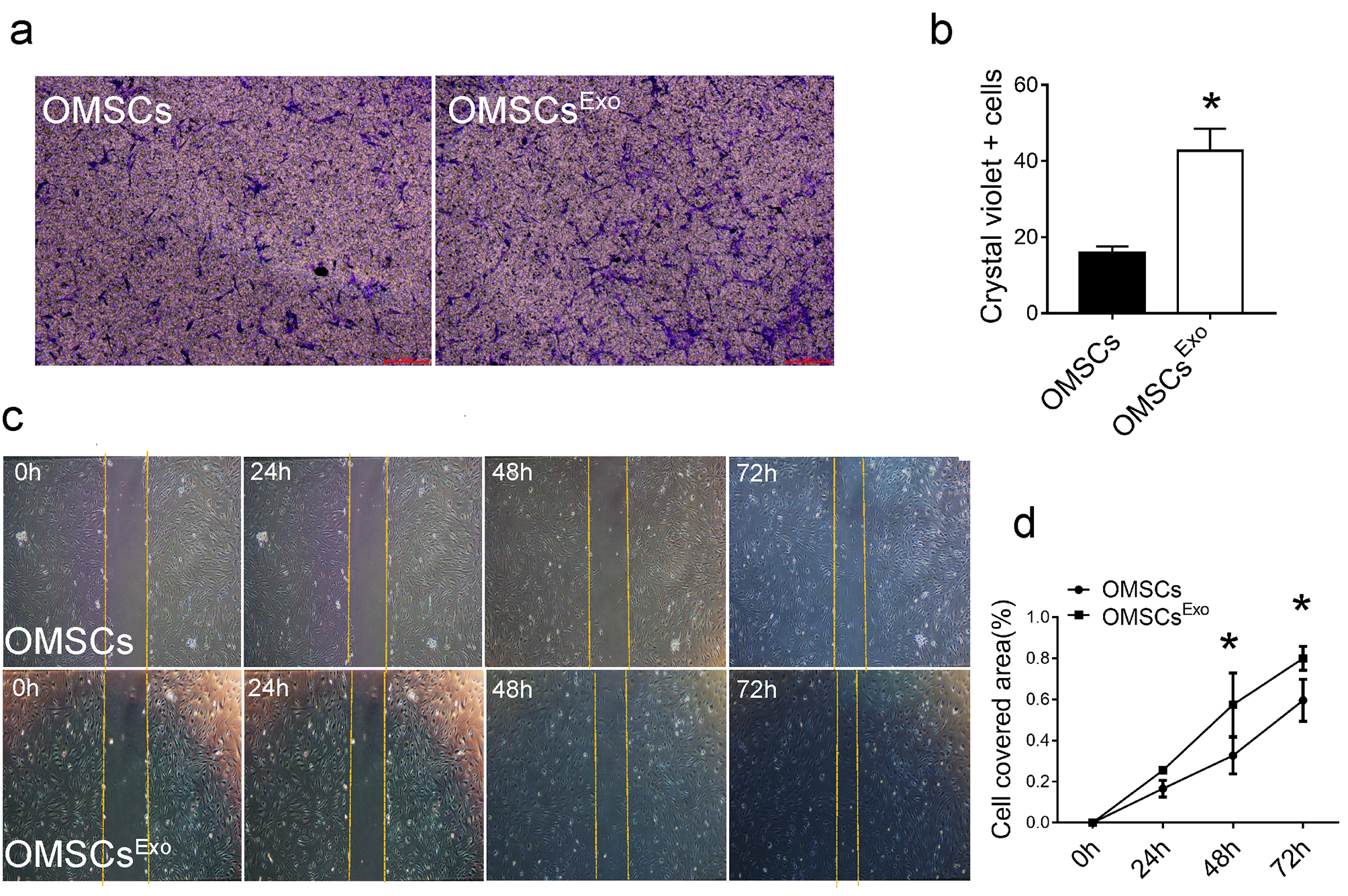

Supplement: Supplementary file 6 — Additional file 6: Figure S3. Migration assay of MSCs. Mobility of OMSCs with or without treatment of ExoUMSCs was analyzed by the transwell assay. Migrated cells were visualized by crystal violet staining. Scratch wound assay was conducted for assessing the migration potential of indicated cells. [file 13287_2020_1782_MOESM6_ESM.tif]

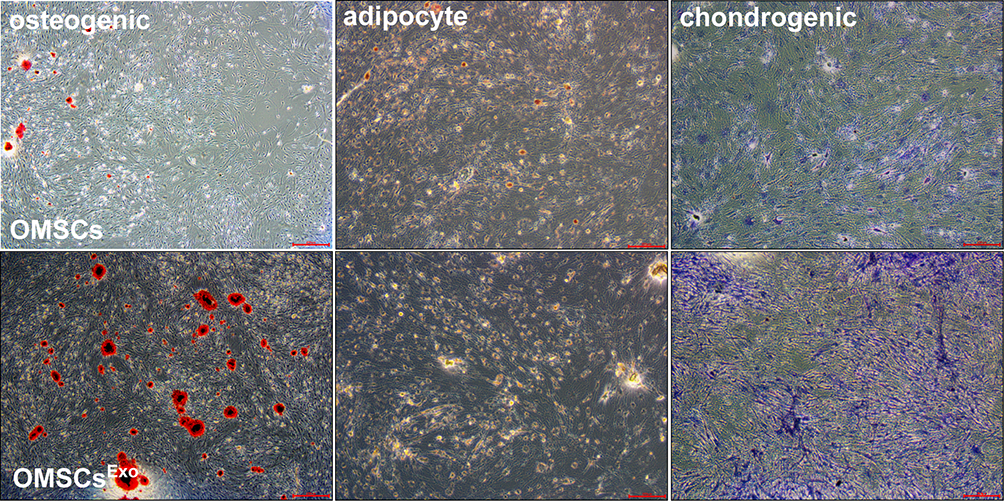

Supplement: Supplementary file 7 — Additional file 7: Figure S4. Differentiation potential of OMSCs after treatment with ExoUMSCs. Representative images of differentiation of OMSCs and OMSCs pretreated with ExoUMSCs into osteocytes, adipocytes and chondrocytes, which was visualized by staining with alizarin red, oil red O, and toluidine blue, respectively. [file 13287_2020_1782_MOESM7_ESM.tif]

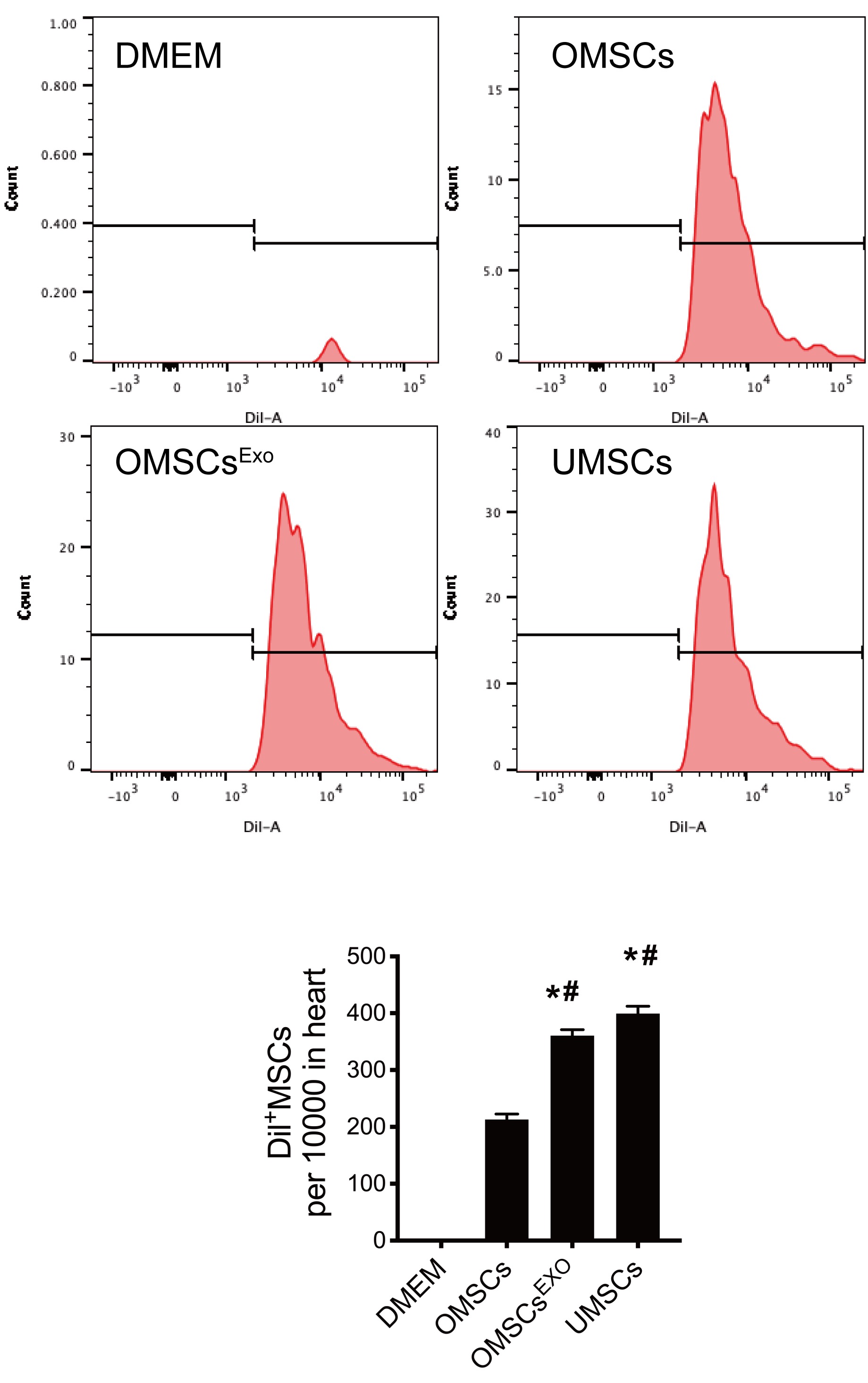

Supplement: Supplementary file 8 — Additional file 8: Figure S5. Cell survival after transplantation after MI. Flow cytometric analysis of Dil positive MSCs injected in the peri-infarct myocardial region after myocardial infarction in different groups. [file 13287_2020_1782_MOESM8_ESM.tif]

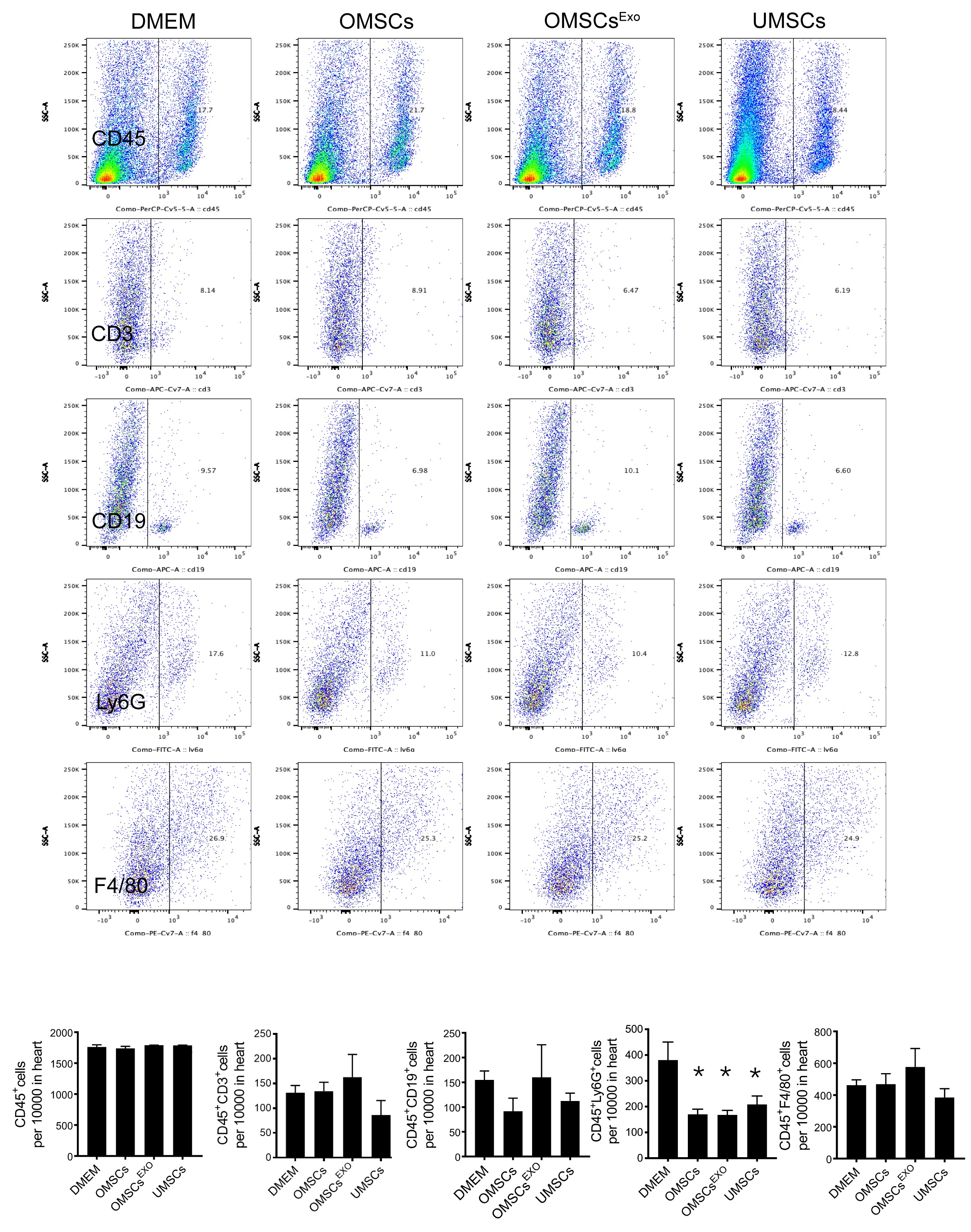

Supplement: Supplementary file 9 — Additional file 9: Figure S6. Immune cells and inflammatory factors expression after MSCs transplantation. Flow cytometric analysis of immune cells including CD3 + B cells, CD19 + T cells, Ly6G + neutrophils and F4/80 + macrophages in heart tissue at day 7 after myocardial infarction in different groups. RT-PCR analysis of inflammatory factors such as IL-1b, Il-6, IL-12, TNFa and MCP-1 in heart tissue at day 7 after myocardial infarction in different groups. [file 13287_2020_1782_MOESM9_ESM.tif]

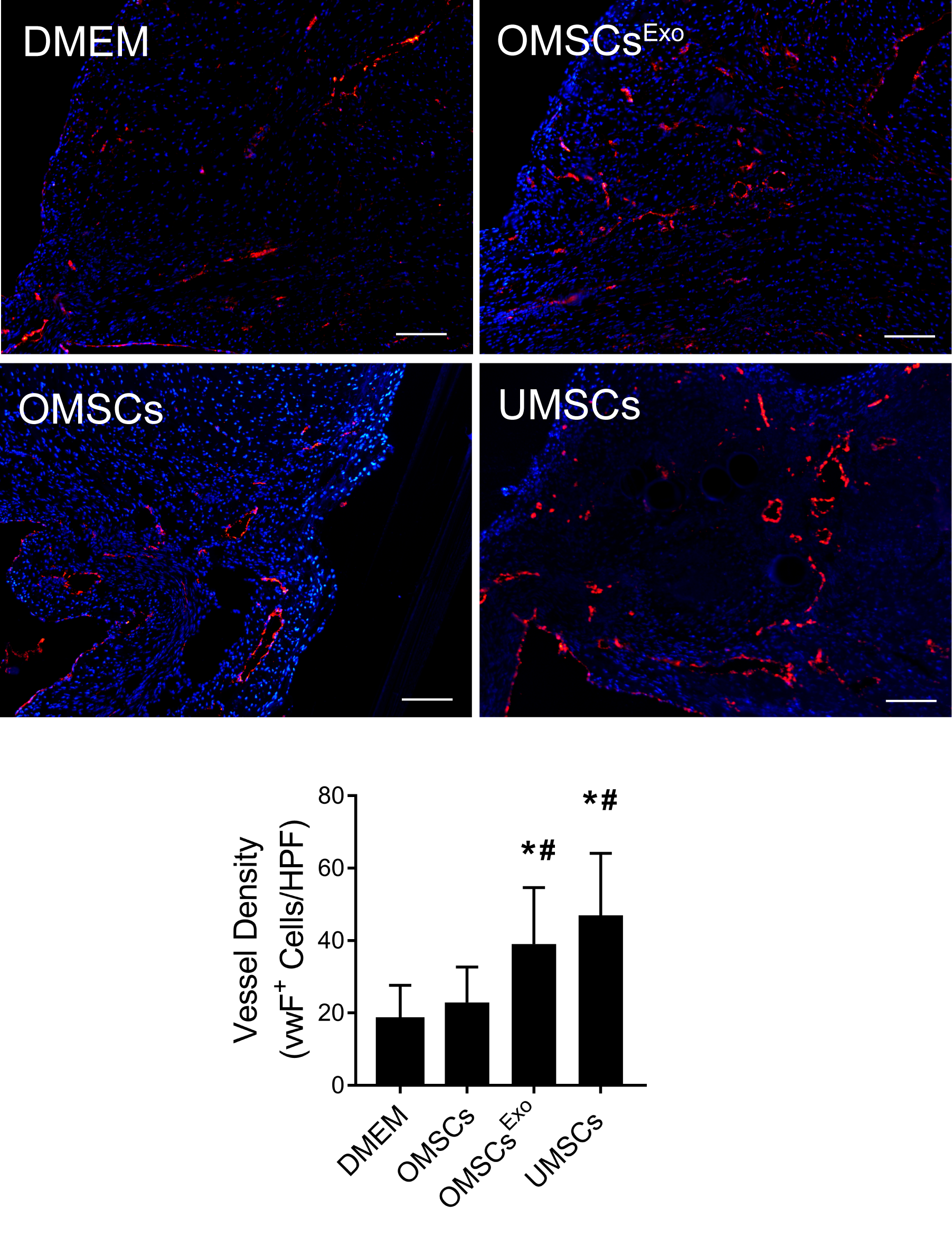

Supplement: Supplementary file 10 — Additional file 10: Figure S7. Effects of MSCs Transplantation on angiogenesis after MI. Representative images of immunofluorescence staining for arterioles using shp against vwF (red) to illustrate matured vessel in the hearts. Scale bar,100 μm. vwF positive cells were quantified per HPF to calculate the matured vessel density in a bar graph. [file 13287_2020_1782_MOESM10_ESM.tif]

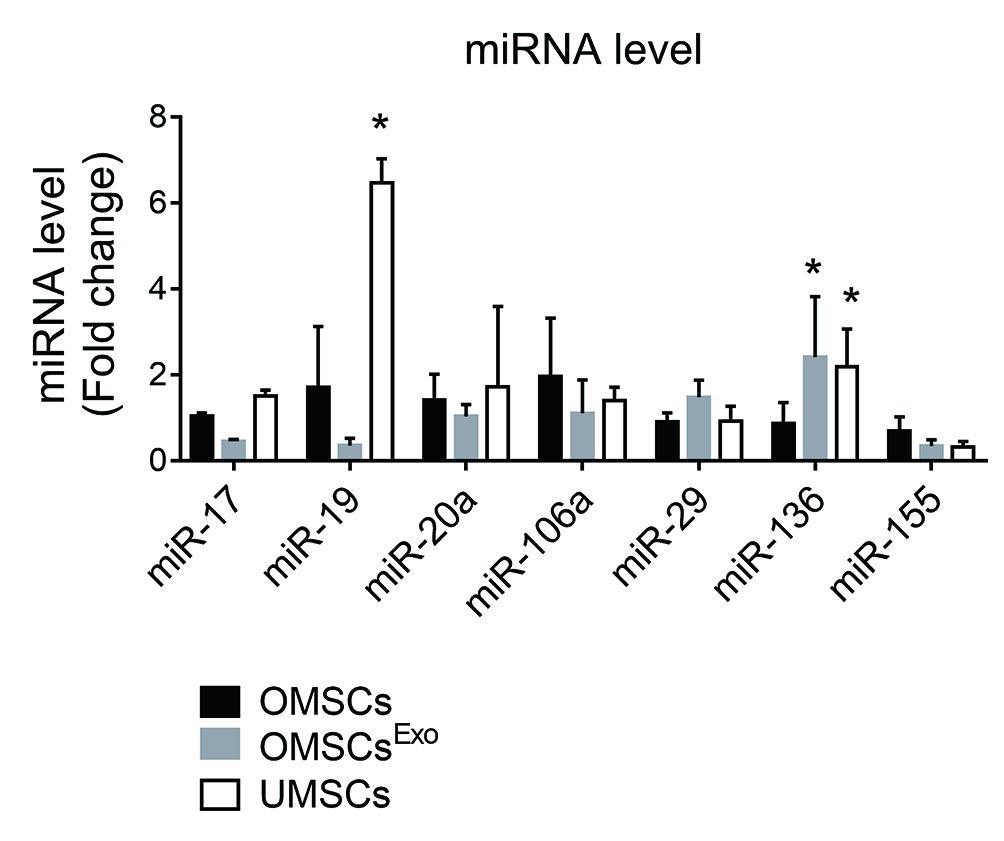

Supplement: Supplementary file 11 — Additional file 11: Figure S8. MiRNAs expression in MSCs. The expressions of miR-17, 19, 20a, 106a, 29, 136, and 155 in OMSCs, OMSCs treated with ExoUMSCs, and UMSCs were assessed by RT-qPCR. U6 was used as an internal reference gene. [file 13287_2020_1782_MOESM11_ESM.tif]

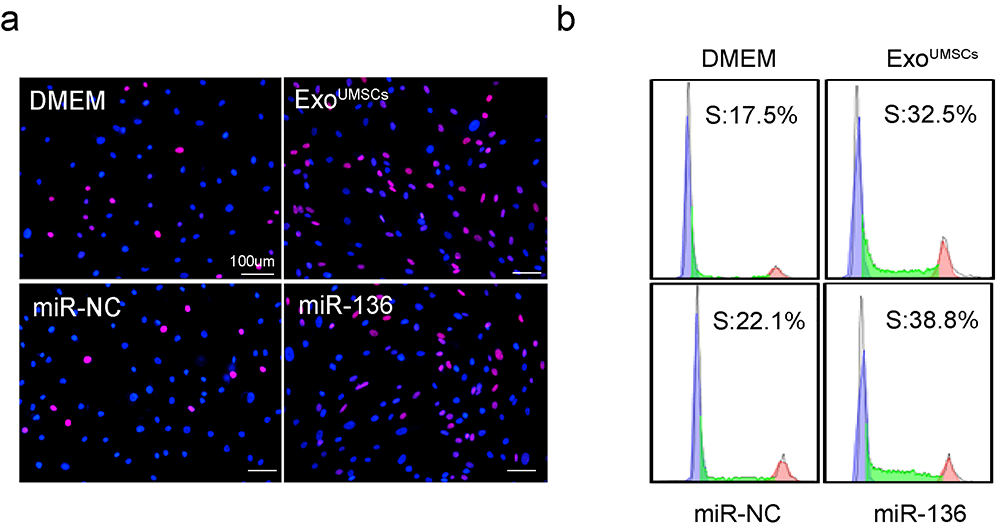

Supplement: Supplementary file 12 — Additional file 12: Figure S9. Detection of OMSCs proliferation and cell cycle after treatment with ExoUMSCs or miR-136. OMSCs which treated with ExoUMSCs, or transfected with miR-136 mimic or miR-NC were analyzed for cell cycle by EdU staining kit and flow cytometry analysis. [file 13287_2020_1782_MOESM12_ESM.tif]

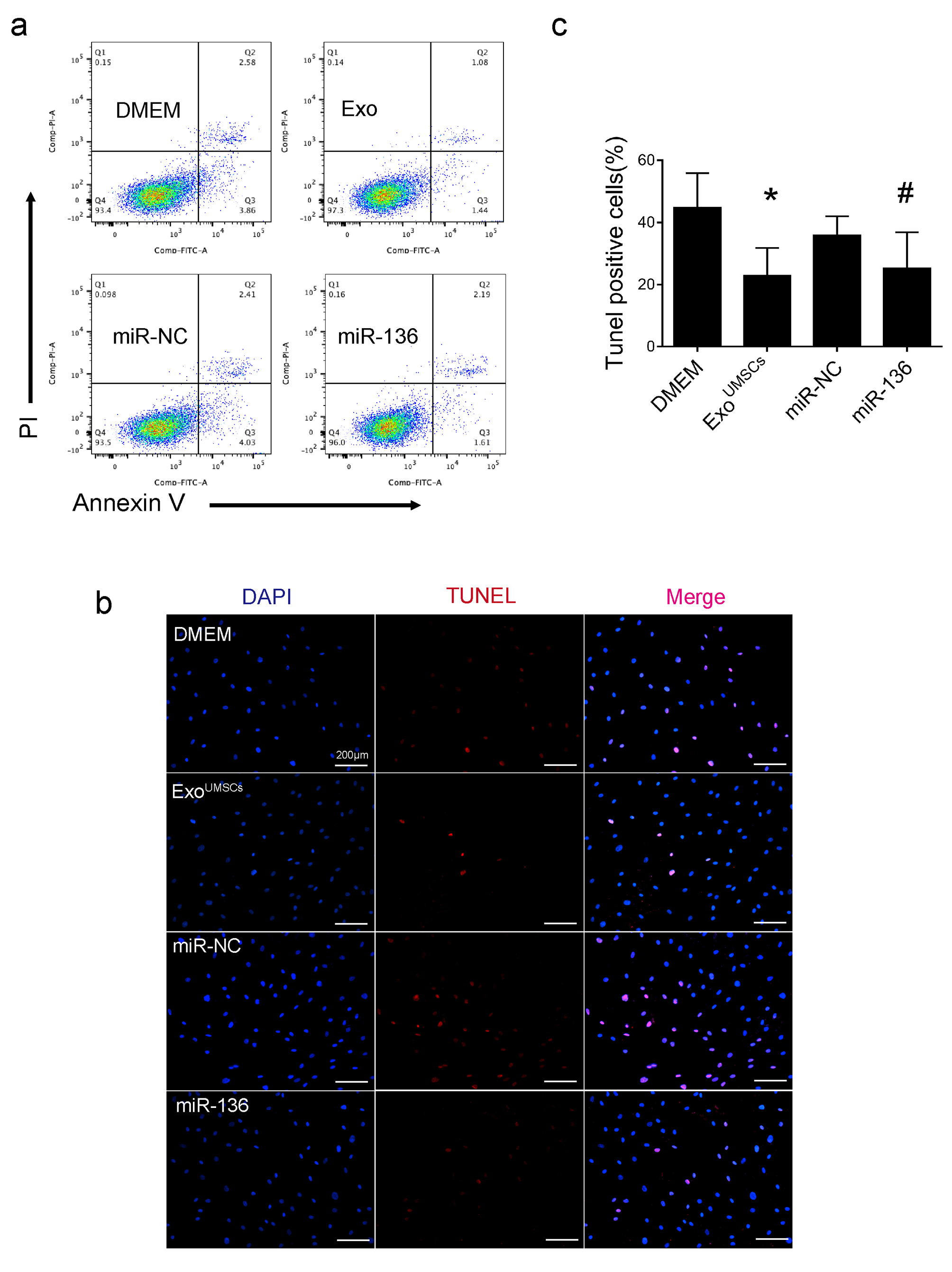

Supplement: Supplementary file 13 — Additional file 13: Figure S10. Apoptosis of OMSCs after transfection of miR-136 or treatment with ExoUMSCs. apoptotic OMSCs were detected by Annexin V/PI staining and TUNEL staining which treated with ExoUMSCs or transfected with miR-136 mimic or miR-NC, and then cultured under hypoxia and serum deprivation conditions. [file 13287_2020_1782_MOESM13_ESM.tif]

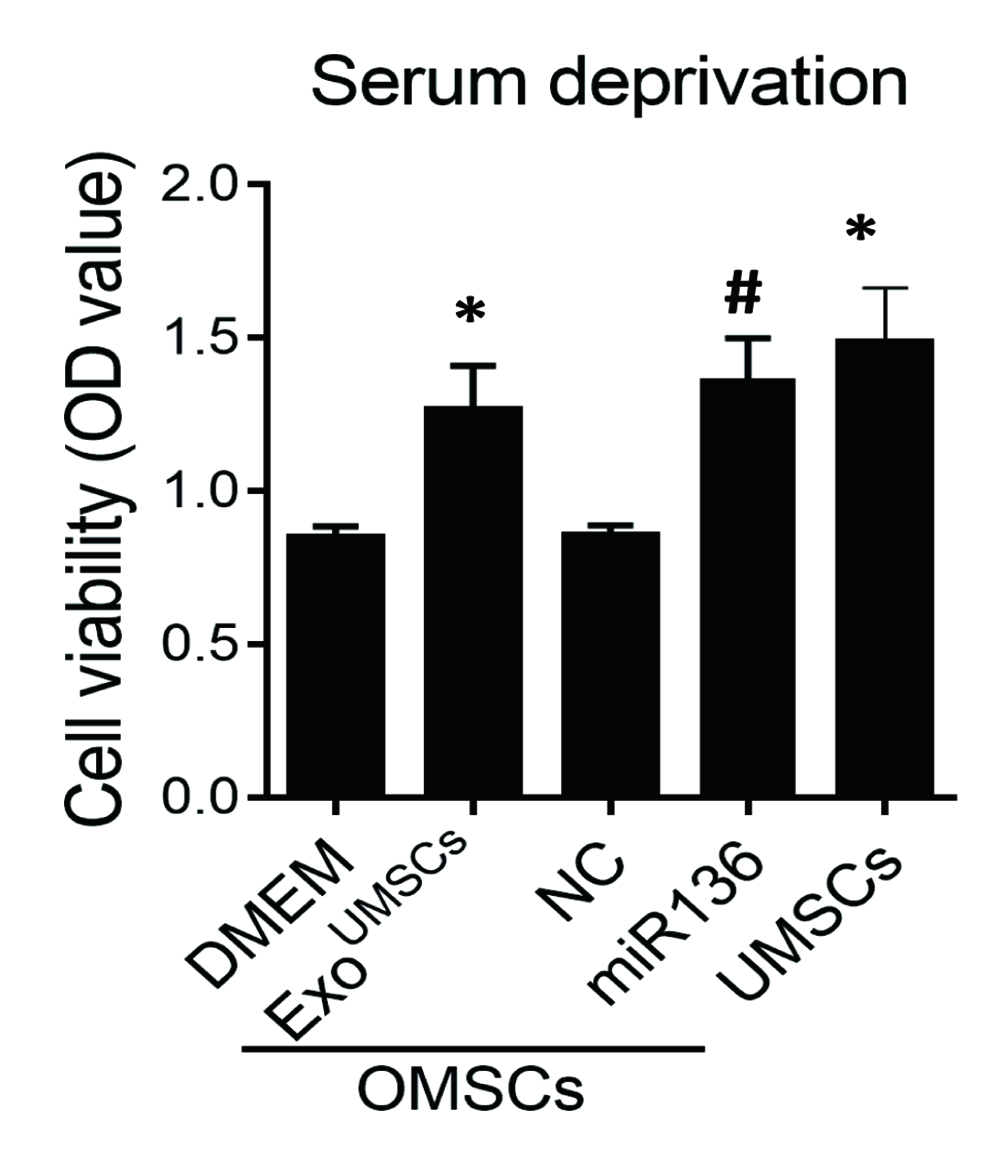

Supplement: Supplementary file 14 — Additional file 14: Figure S11. Quantification of viability of OMSCs. Cell viability of OMSCs with specified treatments was assessed by CCK-8 assay under serum deficiency. [file 13287_2020_1782_MOESM14_ESM.tif]

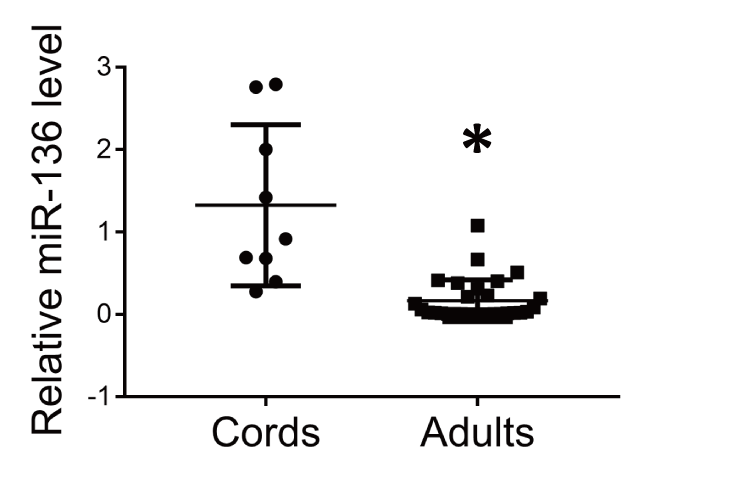

Supplement: Supplementary file 15 — Additional file 15: Figure S12. More miR-136 in cord blood than in adult circulation. Quantification of miR-136 level in serum from adults (n = 29) and cord blood of healthy maternity (n = 9) by real time PCR. U6 was used as an internal reference gene for miRNA. [file 13287_2020_1782_MOESM15_ESM.tif]
